# Co-expression of Skp and FkpA chaperones improves cell viability and alters the global expression of stress response genes during scFvD1.3 production

**DOI:** 10.1186/1475-2859-9-22

**Published:** 2010-04-13

**Authors:** Dave Siak-Wei Ow, Denis Yong-Xiang Lim, Peter Morin Nissom, Andrea Camattari, Victor Vai-Tak Wong

**Affiliations:** 1 Bioprocessing Technology Institute, Agency for Science, Technology and Research (A*STAR), #06-01, Centros, 20 Biopolis Way, 138668, Singapore; 2 School of Biological Sciences, Nanyang Technological University, 637551, Singapore

## Abstract

**Background:**

The overexpression of scFv antibody fragments in the periplasmic space of *Escherichia coli *frequently results in extensive protein misfolding and loss of cell viability. Although protein folding factors such as Skp and FkpA are often exploited to restore the solubility and functionality of recombinant protein products, their exact impact on cellular metabolism during periplasmic antibody fragment expression is not clearly understood. In this study, we expressed the scFvD1.3 antibody fragment in *E. coli *BL21 and evaluated the overall physiological and global gene expression changes upon Skp or FkpA co-expression.

**Results:**

The periplasmic expression of scFvD1.3 led to a rapid accumulation of insoluble scFvD1.3 proteins and a decrease in cell viability. The co-expression of Skp and FkpA improved scFvD1.3 solubility and cell viability in a dosage-dependent manner. Through mutagenesis experiments, it was found that only the chaperone activity of FkpA, not the peptidyl-prolyl isomerase (PPIase) activity, is required for the improvement in cell viability. Global gene expression analysis of the scFvD1.3 cells over the chaperone-expressing cells showed a clear up-regulation of genes involved in heat-shock and misfolded protein stress responses. These included genes of the major HSP70 DnaK chaperone family and key proteases belonging to the Clp and Lon protease systems. Other metabolic gene expression trends include: (1) the differential regulation of several energy metabolic genes, (2) down-regulation of the central metabolic TCA cycle and transport genes, and (3) up-regulation of ribosomal genes.

**Conclusions:**

The simultaneous activation of multiple stress related and other metabolic genes may constitute the stress response to protein misfolding in the scFvD1.3 cells. These gene expression information could prove to be valuable for the selection and construction of reporter contructs to monitor the misfolded protein stress response during antibody fragment production.

## Background

Monoclonal antibodies are widely-used for the diagnosis and treatment of several diseases like cancer and auto-immune disorders. With modern advances in recombinant DNA technology, smaller fragments of these antibodies can be constructed without losing the specificity of their antigen binding [1,2]. Single-chain variable fragment (scFv) is formed by the association of the V_H _and V_L _domains of the antibody with a short polypeptide linker. The smaller size of these scFv fragments allows better tissue penetration leading to improved tumor-targeting [3] and enhanced blood-brain barrier permeability for treatment of neurodegenerative diseases [4]. By far, the most popular system for scFv production is by means of periplasmic expression in *Escherichia coli *[5]. The periplasm of *E. coli *provides a more oxidizing environment than the cytosol, which promotes disulphide bond formation, and the periplasmic space also contains fewer host proteins as compared to the cytoplasm, thus facilitating subsequent purification processes. However, when expression of scFv is high, the increased demand for protein folding could generate an uncharacterized metabolic burden on the cells leading to protein misfolding and aggregation [6].

The periplasmic localization of several protein folding factors and chaperones catalyze the proper assembly and folding of functional scFv antibody fragments [7,8]. Two established periplasmic protein folding factors in *E. coli *are Skp and FkpA. Skp is a key periplasmic chaperone for outer membrane protein assembly in *E. coli *[9] that facilitates proper folding of outer membrane protein intermediates and helps to maintain their solubility [10]. The absence of Skp often leads to protein aggregation in the periplasm, thus reinforcing the importance of Skp as a periplasmic chaperone in *E. coli*. Co-expression of Skp together with scFv fragments in *E. coli *periplasm increased scFv solubility and prevented cell lysis during shake flask cultures [11]. FkpA is another periplasmic protein folding factor that exhibits both peptidyl-prolyl-isomerase (PPIase) and chaperone activities [12,13]. The expression of FkpA alleviated the RpoE-dependant stress response in *E. coli *cells during accumulation of misfolded proteins [14] and it also suppressed the formation of inclusion bodies and promoted proper folding when co-expressed with a folding-defective protein variant [15]. The co-expression of FkpA with scFv significantly improved the latter's soluble and functional expression [16]. Although these protein folding factors are increasingly exploited to improve the soluble expression of recombinant protein products in the periplasm, the detailed impact on host cell metabolism is still not clearly understood.

The 25 kDa scFvD1.3 is a well-characterized antibody fragment against lysoyzme commonly-used as a model for antigen-antibody association studies [17-19]. In this study, we evaluated the overall physiological and global gene expression changes upon Skp or FkpA co-expression. N-terminal and C-terminal mutants of FkpA were also constructed to assess the relative importance of the chaperone and PPIase activities on periplasmic scFv expression and the consequential effect on cell viablity. Although a previous proteomic study using two-dimensional polyacrylamide gel electrophoresis was conducted on F(ab')_2 _antibody fragment-producing *E. coli *[20], this is the first global gene expression study on scFv antibody fragment-producing *E. coli *co-expressing periplasmic chaperones. The aim is to use the physiological and gene expression information to gain insight into important host cell processes such as central metabolism and misfolded-protein stress response in antibody fragment-producing *E. coli*.

## Results and discussion

### Recovery of cell viability in scFvD1.3 cells upon Skp or FkpA co-expression

**Figure 1:**
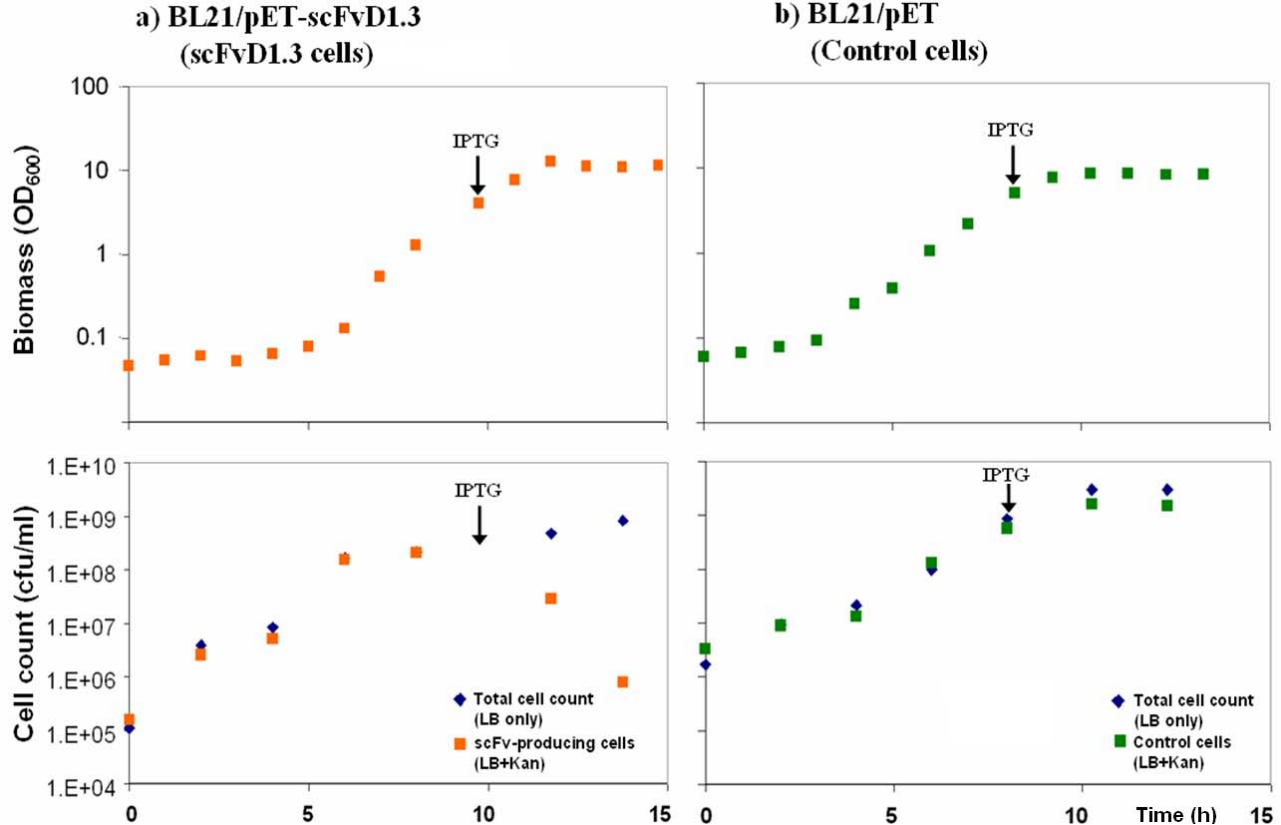
**Growth of (a) scFvD1.3 cells and (b) BL21/pET control cells during 2 L batch cultures**. The point of IPTG induction is indicated by an arrow (↓). Viable cell count of scFvD1.3 cells (orange squares) decreased rapidly after induction.

**Figure 2:**
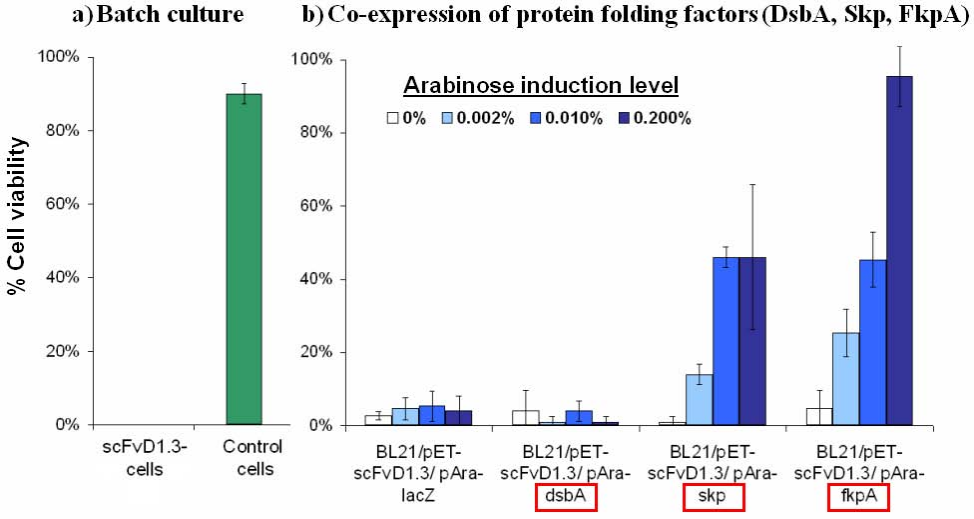
**% Cell viability on LB Kanamycin plates 5 h after induction**. (a) From the batch culture, no scFvD1.3-producing cells could be detected (out of 50 colonies). (b) Co-expression of protein folding factors Skp and FkpA improves % cell viability.

As the anti-lysozyme scFvD1.3 protein contains two internal disulfide bonds, it was expressed in the periplasmic space of *E. coli *where the oxidising environment and disulfide bond enzymes facilitate its functional assembly. *E. coli *BL21 cells carrying the pET-scFvD1.3 or a blank pET plasmid were cultured in 2 L bioreactors to late-log phase before IPTG induction of scFvD1.3 expression. The viable cell count on LB kanamycin (LB+Kan) was seen to decrease drastically upon induction for the scFv cells, indicating a loss of cell viability upon scFv expression (Figure 1). 5 hours after induction, cell viability approached 0% for the scFvD1.3 cells (Figure 2a). In contrast, a higher cell viability of 90% was observed for cells carrying the empty plasmid. The loss of cell viability of the wildtype scFvD1.3 cells upon IPTG induction suggests that the expression of scFvD1.3 leads to cell toxicity or elevated metabolic burden.

**Figure 3:**
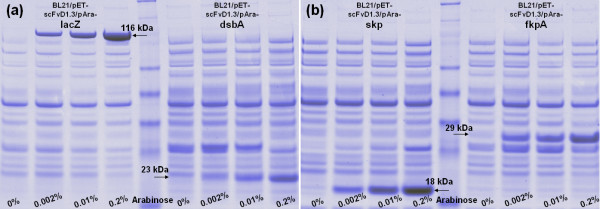
**SDS-PAGE analysis of strains co-expressing LacZ, DsbA, Skp, FkpA**. The intensity of protein bands corresponding to (a) LacZ (116 kDa), DsbA (23 kDa) and (b) Skp (18 kDa), FkpA (29 kDa) increased for strains co-expressing these proteins with increasing arabinose induction.

To test if the co-expression of protein folding factors with scFvD1.3 could improve cell viability, expression vectors for three folding factors (DsbA, Skp and FkpA) under the control of an arabinose-inducible promoter were co-transformed with the pET-scFvD1.3 vector into the scFvD1.3 cells. DsbA is a disulfide oxidoreductase catalyzing disulfide bond formation in *E. coli *periplasm [21,22]. Skp is a periplasmic chaperone assisting outer membrane protein assembly, and FkpA is a periplasmic protein folding factor with both PPIase and chaperone functions. Co-expression of LacZ was included as a negative control. SDS-PAGE of total cellular proteins in the four co-expressing strains (Figure 3a &3b) showed a gradual increase in the intensity of bands corresponding to the expected sizes of LacZ, DsbA, Skp and FkpA, indicating an actual increase in the expression when induced with increasing arabinose concentration. Upon the induction of these folding factors with up to 0.2% arabinose, the cell viability of the Skp and FkpA co-expressing cells increased to 46% and 95% respectively from an uninduced value of less than 5% (Figure 2b). On the other hand, co-expression of either LacZ control or DsbA both showed negligible effect on cell viability. This shows that the co-expression of Skp or FkpA improved cell viability of scFvD1.3 cells in a dosage-dependent manner.

**Figure 4:**
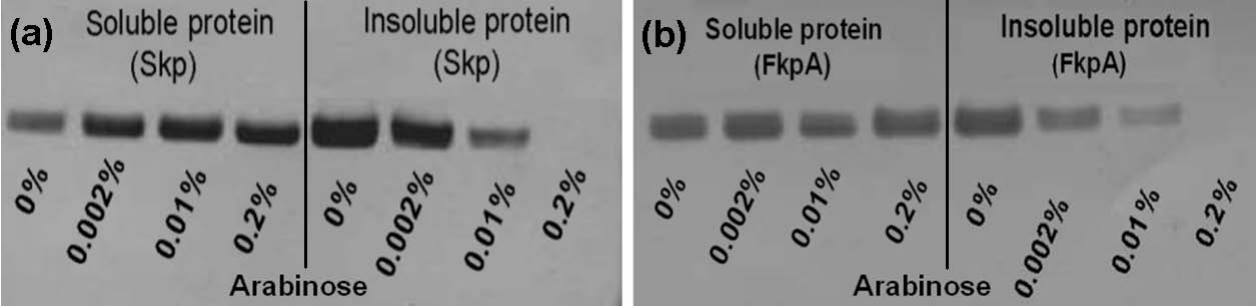
**Coexpression of protein folding factors FkpA and Skp reduced the amount of insoluble proteins**. From Western blot, the amount of insoluble scFvD1.3 proteins after sonication reduced with increasing arabinose induction of (a) Skp or (b) FkpA.

As Skp and FkpA are both molecular chaperones with general ability to refold misfolded proteins and prevent their aggregation into insoluble inclusion bodies in the periplasm, the soluble and insoluble scFvD1.3 yields in the Skp/scFvD1.3 and FkpA/scFvD1.3 cells were examined with Western blot. For the Skp/scFvD1.3 cells, a decrease in insoluble scFvD1.3 protein content was observed with increasing Skp co-expression (Figure 4a). Correspondingly, an increase in soluble scFvD1.3 yield was observed. Likewise for the FkpA/scFvD1.3 cells, a reduction in insoluble scFvD1.3 protein content with increasing FkpA co-expression was shown (Figure 4b). Taken together, these results suggest that the higher cell viability of cells co-expressing Skp or FkpA with scFvD1.3 could be related to their increased chaperone-mediated capacity to avert protein aggregation leading to lower accumulation of insoluble scFvD1.3 proteins and reduced metabolic stress. Additionally, the effect of co-expressing both Skp and FkpA together on scFvD1.3 production and cell viability were also examined. When both folding factors were simultaneously expressed in the scFvD1.3 cells using 0.01% arabinose induction (Table 1), the scFvD1.3 yield and cell viability increased approximately 3 fold over the lacZ control. However, these improvements in scFvD1.3 yield and cell viability are not higher than the expression of Skp or FkpA individually (between 8-10 fold). This indicates that the co-expression of Skp and FkpA together may not have a synergistic effect on scFvD1.3 production and cell viability.

**Table 1:** Co-expression of Skp and FkpA on scFvD1.3 yield and viability

	scFvD1.3 yield		scFvD1.3 cell viability	
Protein(s) co-expressed	(mg/L)	Fold increase	(%)	Fold increase
LacZ (control)	5.4 ± 1.5	-	5.3 ± 4.2	-
Skp	51.2 ± 6.9	10×	46.0 ± 2.8	9×
FkpA	42.6 ± 6.1	8×	45.3 ± 7.6	9×
Skp + FkpA	18.3 ± 2.7	3×	18.0 ± 2.8	3×

While the co-expression of Skp or FkpA alone at 0.01% arabinose resulted in a 8-10 fold increase in scfvD1.3 yield and cell viability over the LacZ control, the co-expression of Skp and FkpA together led to only a 3 fold increase.

### The N-terminal chaperone activity of FkpA is required for cell viability recovery

**Figure 5:**
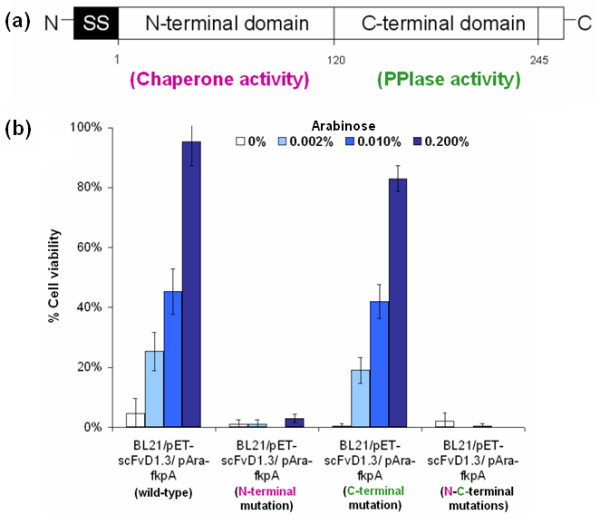
**Evaluating the functional domains of FkpA**. (a) The N-terminal and the C-terminal of FkpA are responsible for the chaperone and the PPIase activity respectively. (b) Removal of the chaperone activity by N-terminal mutations abolished the beneficial effect of co-expressing FkpA on cell viability.

The mature FkpA consists of 245 amino acid residues with two functional domains (Figure 5a), the N-terminal and C-terminal domain [13]. The N-terminal domain, consisting mainly of α-helices, contributes to the dimerisation and chaperone activity of FkpA [15]. On the other hand, the C-terminal domain, being able to catalyze slow refolding *cis-trans *isomerisation reactions in proline-containing proteins, is responsible for the PPIase activity [23]. As the chaperone-like activity of FkpA is independent of its PPIase activity [23,24] and only requires the presence of the N-terminal domain [15], the deletion of the amino acid 3 to 96 at the N-terminal of FkpA was shown to abolish the chaperone activity. Conversely, the C-terminal amino acid substitution of Ile at 174 to Ser and Gly at 176 to Ser was shown to diminish the PPIase activity of FkpA [15].

To assess if both the chaperone and the PPIase activities of FkpA are essential for the improvement in cell viability, the N-terminal, C-terminal and N-C terminal mutants of FkpA were constructed and cloned under the control of an arabinose inducible promoter. Mutation at the N-terminal was created by overlap PCR removal of 93 amino acid residues in the chaperone domain, while the C-terminal mutation was generated using site-directed mutagenesis of the key active sites in the PPIase domain. From co-expression experiments, it was found that only the N-terminal and N-C terminal mutations abolished the positive effect of FkpA co-expression on cell viability (Figure 5b). On the other hand, co-expression of the C-terminal mutant form lacking PPIase activity showed a comparable phenotype to the wild-type FkpA. Altogether, it appears that the protein refolding activity (chaperone) of FkpA rather than its assembly activity (PPIase) plays a more crucial role in overcoming metabolic stresses from protein misfolding and improving cell viability during scFvD1.3 production.

### Microarray transcriptional analysis of chaperone co-expressing scFvD1.3 cells

As the co-expression of chaperones Skp or FkpA markedly improved protein solubility and cell viability in scFvD1.3 cells, it is of interest to further characterise the overall metabolic changes in these cells. Using Affymetrix *E. coli *genome 2.0 arrays, global transcriptional changes in the scFvD1.3 cells were compared to the chaperone co-expressing Skp/scFvD1.3 and FkpA/scFvD1.3 cells. Bioreactor cultures of scFvD1.3, Skp/scFvD1.3 and FkpA/scFvD1.3 cells were cultured under identical conditions and samples for microarray were collected three hours post-induction (with 0.2% arabinose and 20 μM IPTG). Statistical expression analysis of signal intensity on individual arrays revealed that between 74-86% of 4070 *E. coli *probe sets on each array were significantly detected as present. Subsequent comparison expression analyses of the scFvD1.3 versus the Skp/scFvD1.3 or the FkpA/scFvD1.3 cells were made. After filtering for genes commonly up- or down-regulated by 1.5 fold or more (change p-value ≤ 0.0025 or ≥ 0.9975) from two biological replicates, a total of 268 up-regulated genes and 355 down-regulated genes were obtained (Table 2; Additional file 1, Additional file 2, Additional file 3, Additional file 4). These regulated genes were classified according to their functional catagories to identify the gene expression trends.

**Table 2:** Differentially-regulated genes for scFvD.13 cells over the Skp/scFvD1.3 or FkpA/scFvD1.3 cells

	scFvD1.3 vs Skp/scFvD1.3		scFvD1.3 vs FkpA/scFvD1.3	
Functions	Up-regulated	Down-regulated	Up-regulated	Down-regulated
**Heat shock stress response, protein folding and related**	***clpS****dnaJ grpE hslR lon ybbN*	***asr skp***	*clpS ****clpP clpX dsbC ****dnaJ fkpB ****grpE hslO hslR hslU hslV hspQ htpX ibpA lon ppiC prc secA secY tatB ****ybbN*	*dps ****fkpA ****sodC uspB uspE*

**Energy metabolism**	*cydA cydB*	*frdB frdC nuoA nuoC nuoE nuoF ****nuoG****nuoH nuoI nuoJ nuoK nuoL ****nuoM***	***ldhA***	*frdB*
**TCA cycle and other carbon metabolic pathways**	*gcd*	***acs aldA****araD arnT ****astD****fucI fucU gcvP glcC glgX maeB mazG mtlD ****nagE****rffH sdhA sdhB sdhD sucA sucB sucC sucD*	*aceE ****araB****araD ****gcd***	***aceB****aceK acnA ****acs aldA astD fbaB****fucI fumA fumC gatY gatZ ****glcB gluD glcF****glk gltA maeB ****manX****mdh mtlD pckA poxB rbsD sdhB sdhD sucD tktB yiaG yjgB*
**Transport**	***proP****tauA*	*artQ dppB dppC cycA ****fadL fucK****galP glnH glnP ****glnQ gltJ****gltK gltL gntP gntT gntU hcaT hisQ hisM hisP ****mglA****mglB ****mglC****mgtA mtlR nanT nhaA pstA pstS rbsA rbsC rbsK rbsR sdaC sstT tsx uxuB ****yjcG***	***araE aqpZ arsB****dusB ****potA sotB****nepI ****proP tauA tolA tolR****ybeX ygeD*	***argT****chbB cycA ****fadL****gabP ****galP galS glcA glcG****glpT glnH glnP glnQ ****gltI gltJ gltK****gltL gntP ****lamB****manY manZ ****mglA****mglB mglC oppA ****oppB****putP rbsA rbsC rbsK srlA srlB tsx ugpA ****yjcG****yjdN yhiP ****yphF***

**Amino acids**	*cysW ****ilvC****sdaA*	***astB****astC ****astE****beta carA dadX gabT hisA leuB leuD sdaB tnaA ubiH*	***alr argH aroF aroK cysM cysW emrD iaaA ilvC****sdaA ****thrA thrB ygeA***	***aceA****aphA ****astB astC astE****dadA dadX ****gabT****katG melA pepE putA ****tnaA tnaC***
**Nucleic acids, nucleotides and cofactors**	*menB ribH rrmJ topA*	***fadA****polB ppx purB purL ndk rrmA ubiB*	***cmk cobS cyaA der dnaG gpp gsk guaB gyrB hflX holC****infB menB ****menC****mfd ****obgE****proW priB ****prlC queA rdgB****recF ****ribH****rimM rluA ****rnb rnc rng rnhB****rplI ****rplY rpmB****rpsK ****rpsP rrmJ srmB thrC****topA ****truB xerD****yjgP*	*allA elaB ****fadA****rsd ****tnaB****yaaF ybaE ybeK yjbJ*
**Lipid and cell wall-related**	*miaA mltD mutM*	*ddlB fadD fadE ****fadH fadI fadJ ftsI****mepA mraY mrdA murC ****murD****murG yeiU yqeF yrfF*	***cutE fabD glmM lolC lpxP miaA mltD mrcA mrdA msbA****mutM ****prfB rlpB yfgB***	*anmK blc csiE ****ecnB****erfK ****fadD fadE fadH fadI fadJ****hdfR ****ybaW****yqeF*

**Transcription and translation related**	*rplR rpmC rpmD rpmI rpsN rpsQ*		***deaD fusA hisS****leuS ****glnS ileS****lspA ****nusA pheS pheT prfC****rbfA ****rluB rplB****rplC rplD ****rplE****rplF ****rplJ rplK rplL rplM****rplO rplP rplR ****rplS rplT rplU****rplV ****rplW rplX rpmA rpmC rpmD rpmI rpmJ rpoA rpoB****rpoC ****rpoD****rpsA rpsB ****rpsC****rpsD rpsE ****rpsG****rpsH ****rpsI****rpsJ ****rpsM****rpsN rpsQ ****rpsR****rpsS trmD ****valS***	*sra*

**Expression regulators and cell division**	*cbl Spf yceP ****ycfR zntR***	*araC ****csiD****flhC ftsL* *ftsQ galS glnG hcaR malT ****metH****mraW pstB ****soxS****sraH*	*arsR ****csdA cspA cysK****emrR ****flgM****fis ****glpE****hflB hflC ****hflK narP rnxG rplA****rplQ ****rpmE spf yceP ycfR zntR***	*bolA ****csiD****cspE ****cyaR****feaR fruR fucR glcC ****hcaR****hexR hokB ldrD melR minD minE mokB omrA sokB yidF yeiL*
**Signalling and motility**		*cspD cstA envZ flgD ****flgG****flgH flgI flgJ ****flgK*** *flgL purD purH*	*flgC ****flgG****hepA ****htpG****typA*	*atoD ****cspD****cstA ddpX ompW yjdI ****yjjM***
**Unknown or other miscellaneous functions**	***cysD****yceD yciS ycjF ycjX ydhQ ydjN yeeD yeeE ****yhdN****yoaE ****yohJ***	*glgS rffA rffC spr ubiA visC ybhQ ycdN ydhC yeeI yehU yhdP yhhN ****yjcH****yphF ytfS*	***araJ****cysD ****cysH cysJ ecfK folD gntY mdtD mdtH mscS psiE****yaeL ****yafD ybeA****ybeD ****ybeY ybeZ ybjX****yceD ****yciM yciS ycjF ycjX****ydhQ ****ydjN yebC yeeD yeeE yfcJ yfgM ygiQ yhcN yhdN****yhhS ****yncJ yohJ yrbD***	*abgA ****aldB bfr****ddpA dkgA ecfJ ****gabD glgS****msrB nanE ****osmC****osmY psiF ribB rof ryeA ****sraH ssnA****ubiA xdhB yahK ****yahN****yaiY yaiZ ybaY ybhQ ybjP ****ycaC yccJ ycgB****ycgK yciI ydcL ydcS ydcU ydcW yddP ydeN yeaQ yeaT ****yebV****yebW yedX ****yeeI yeiT****ygaM ygcO ****ygdI****ygfJ yggE yghZ ygjR yhaH yifE yihW ****yncB****yniA yodB yohF ****yphA yphG****yqjD yqjK ygeV*

Comparison expression analysis of the wildtype scFvD1.3 over the chaperone co-expressing Skp/scFvD1.3 or FkpA/scFvD1.3 cells with Affymetrix GCOS version 1.4. Commonly up- or down-regulated genes (change p-value ≤ 0.0025 or ≥ 0.9975) from two biological replicates are categorized according to their metabolic functions (genes with fold changes of between: (1) 1.5-3 fold are *in italics *only, (2) 3-4 fold are ***bold***, and (3) above 4 fold are ***bold and underlined***).

### Heat shock stress response, protein folding and related processes

Extensive misfolding and aggregation of proteins during heterologous protein production is known to induce a metabolic stress response characterized by the up-regulation of a number of heat shock proteins [25,26]. In line with the observation of increased amount of insoluble proteins in the scFvD1.3 cells without chaperone co-expression, 9 classical heat shock proteins [27] were up-regulated (*dnaJ, grpE, ibpA, clpP, clpX, lon, hslU, hslV, htpX*) in these cells. Several of these upregulated heat shock proteins are cytoplasmic molecular chaperones aiding protein folding and aggregate disassembly in *E. coli*. Co-chaperones DnaJ and GrpE, which are partners of the major Hsp70 DnaK chaperone, are involved in the recognition of misfolded substrates and release of DnaK from the substrate-chaperone complex [28,29]. IbpA is a small heat shock protein first reported to be tightly associated with inclusion bodies during heterologous protein production [30]. It was subsequently found to be a holding chaperone that assists protein folding by stabilizing unfolded protein intermediates during periods of severe stress [31].

In addition to chaperone-assisted refolding, a second metabolic strategy used by *E. coli *to eliminate misfolded proteins is by means of protein degradation pathways. As much as 80% of protein degradation in *E. coli *occurs via two main protease systems: Clp and Lon [32,33]. Interestingly, 3 of the up-regulated genes (*clpP, clpX, lon*) were related to these protease systems. ClpP is a heat shock-regulated serine protease that is part of several major protease complexes including ClpAPX and ClpXP [34]. ClpX, a chaperone responsible for stimulating the ATP-dependent recognition and degradation of abnormal proteins, also serves as a substrate-specificity modulator and activator for ClpAXP and ClpPX complexes. ClpS is another specific modulator for the ClpAP protease complex also found to be up-regulated in the scFvD1.3 cells [35]. Other than Clp family of protease complexes, Lon is the other major protease responsible for the degradation of misfolded proteins and the prevention of protein aggregation [36,37]. In line with its role as a major intracellular protease, strains deficient in Lon exhibited lower rate of abnormal protein degradation compared to the wildtype [38]. Additionally, the HslUV protease complex, which is able to functionally substitute the Lon protease under some conditions, also contributes to overall protein degradation [39]. Both the ATPase component of the HslVU protease (encoded by *hslU*) and the peptidase component (encoded by *hslV*) showed significant up-regulation [40]. The last classical heat shock protein, HtpX, is a putative membrane protease. HtpX expression is induced by high temperature [41] and coordinated by the CpxAR two-component regulatory system that senses and responds to misfolded proteins within the periplasmic space [42].

Other up-regulated genes related to protein folding or degradation include *dsbC, tsp, fkpB, ppiC & hslR*. DsbC is a periplasmic disulfide oxido-reductase required for isomerization and rearrangement of disulfide bonds of periplasmic proteins [43]. The transcription of DsbC is activated by the RpoE regulatory pathway in response to various cell envelope stresses including the accumulation of misfolded periplasmic proteins [44]. Tsp is a periplasmic serine protease that digests unstably folded proteins at several sites with broad primary sequence specificity [45,46]; it also recognizes and digests misfolded cytoplasmic and periplasmic proteins tagged by the SsrA protein quality control system [47]. FkpB and ppiC are both peptidyl-prolyl cis/trans-isomerases catalyzing the cis-trans-isomerization of proline-containing peptide bonds [48,49]. Finally, the *hslR *gene product (Hsp15) is an abundant protein possibly involved in recycling free 50S ribosomal subunits that still carry nascent polypeptide chains abandoned during protein translation [50,51].

On the whole, the concurrent activation of multiple stress genes may constitute the misfolded protein stress response in the scFvD1.3 cells. It was noted that many of the upregulated stress genes encode for cytoplasmic chaperones or proteases rather than periplasmic ones (except for *dsbC *and *tsp*). As it is possible for overproduced foreign protein to obstruct export sites and prevent proper translocation of other cellular proteins [52], we proposed that one of the bottlenecks for scFvD1.3 production could be in the periplasmic translocation step. Consistent with this proposition, it was reported that misfolded IPTG-induced scFv was not released from periplasm after osmotic shock showing that they either associate with the inner membrance or remain within the cytoplasm [53]. Moreover, the cytoplasmic accumulation of unsecreted proteins (from the overproduction of export-defective proteins) also led to a two- to fivefold increase in the synthesis of heat shock proteins [26].

### Bioenergetics, carbon metabolism, transport

The respiratory electron transport chain of *E. coli *contains two distinct membrane-bound NADH dehydrogenases (NDH-1 and NDH-2) both catalysing the oxidation of NADH [54]. The subsequent transfer of electrons via ubiquinone to one of the two main terminal oxidase complexes (cytochrome bd and cytochrome bo) generates a proton motive force, which could be used as an energy source driving ATP synthesis or active transport of nutrients [55]. The NADH dehydrogenase complex I or NDH-1 consists of 14 subunits encoded by *nuoA *to *nuoN *and the deletion of any of the *nuo *genes leads to a complete loss of activity in the membrane [56]. In the Skp/scFvD1.3 cells, 11 of the 14 *nuo *genes of NDH-1 were up-regulated. However, the *cydA *and *cydB *genes encoding the only two subunits of cytochrome bd were down-regulated in these cells (Table 2). Additionally, up-regulation of the *frdB *and *frdC *genes encoding for two separate subunits of fumarate reductase (a terminal electron acceptor under anaerobic or fermentative conditions) was observed [57]. These results seem to suggest that the scFvD1.3 cells could be utilizing different pathways for energy production relative to the Skp/scFvD1.3 cells.

The tricarboxylic acid (TCA) cycle is a central metabolic pathway crucial for the generation of metabolic precursors as well as several high-energy molecules like ATP and NADH [58]. In agreement with the observed lower cell viability of the scFvD1.3 cells, it was noted that the gene expression of many TCA enzymes was down-regulated in these cells relative to the Skp/scFvD1.3 cells (*sdhA, sdhB, sdhD, sucA, sucB, sucC, sucD*) or the FkpA/scFvD1.3 cells (*acnA, fumA, fumC, gltA, mdh, sdhB, sdhD, sucD*). Citrate synthase (encoded by *gltA*) catalyzes the first step in the TCA cycle that feeds acetyl-CoA into the pathway by converting acetyl-CoA to citrate. The product of *acnA*, which catalyzes the second and third steps in TCA, is an aerobic stationary-phase aconitase that is specifically induced by iron and redox-stress [59]. Other downregulation of enzymes responsible for the subsequent TCA reactions included: α ketoglutarate dehydrogenase (*sucA, sucB*), succinyl-CoA synthetase (*sucC, sucD*), succinate dehydrogenase (*sdhA, sdhB, sdhD*), fumarate reductase (*fumA, fumC*), and malate dehydrogenase (*mdh*). The overall down-regulation of TCA cycle enzymes suggests that, compared to the chaperone co-expressing cells, the scFvD1.3 cells could have lower capacity for the generation of metabolic energy.

Other genes related to the TCA cycle include: the NADP-linked malic enzymes gene (*maeB*) which catalyzes the interconversion of TCA intermediates malate and oxaloacetate; acetyl-CoA synthetase gene (*acs*) which has an anabolic scavenging function that converts acetate from the medium into acetyl-CoA that feeds into TCA cycle [60]. *maeB *and *acs *were also downregulated in the scFvD1.3 cells. In addition, consistent with the notion of a lower capacity for energy generation of the scFvD1.3 cells, a number of transport genes catalysing the uptake of alternative carbon sources (*cycA, fadL, galP, glnH, glnQ, gltJ, gltK, gltL, gntP. rbsA, rbsC, rbsK, yjcG*), as well as several genes related to amino acid (*astB, astC, astE, dadX gabT, tnaA*), nucleotide (*purB, purI*) and fatty acid (*fadD, fade, fadH, fadI, fadJ*) metabolism, were also down-regulated.

### Transcription and translation, expression regulators and signalling processes

Related to transcription, three genes (*rpoA, rpoB, rpoC*) encoding for the core enzyme of *E. coli *RNA polymerase showed up-regulation in the scFvD1.3 cells over the FkpA/scFvD1.3 cells. In addition, the *rpoD *gene encoding for the primary sigma factor 70 during exponential growth was also upregulated. Sigma factor 70 enables the RNA polymerase core enzyme to recognise and coordinate the transcription of housekeeping genes during cell growth [61]. Related to translation, the expression of several 50S (*rplB, rplC, rplD, rplE, rplF, rplJ, rplK, rplL, rplM, rplO, rplP, rplR, rplS, rplT, rplU, rplV, rplW, rplX, rpmA, rpmC, rpmD, rpmI, rpmJ*) and 30S (*rpsA, rpsB, rpsC, rpsD, rpsE, rpsG, rpsH, rpsI, rpsJ, rpsM, rpsN, rpsQ, rpsR, rpsS*) ribosomal protein genes were found to be up-regulated in the scFvD1.3 cells. The ribosomal proteins form the bacterial ribosome that initiate protein synthesis according to information encoded in the mRNAs and their expession are coordinately-regulated in response to the rate of growth and protein synthesis [62,63]. Related to translation as well, the upregulation of seven tRNA synthetase genes (*hisS, leuS, GlnS, ileS, pheS, pheT, valS*) responsible for covalent linkage of various amino acids to their specific tRNA molecules was also seen [64]. Other upregulated genes related to the ribosome function and processing include: *deaD *- which assembles the large 50S ribosomal subunit [65], *fusA *- an elongation factor that facilitates the translocation of ribosome along the mRNA [66], *prfC *- a GTPase which facilitates the release of the growing polypeptide chain at stop codons [67], and *rbfA *- a 30S ribosomal subunit associated protein required for the efficient processing of the 16S rRNA [68]. The results suggest that the scFvD1.3 cells could be actively synthesizing more host proteins such as chaperones and proteases in response to the metabolic stress compared to the FkpA/scFvD1.3 cells. Finally, several genes encoding for transcriptional regulators, cell division, signalling and motility proteins also exhibited differential-regulation. The former includes *flhC*, the principal regulator of flagellum biogenesis and swarming migration [69] and the *fruR *(also known as *cra*) pleiotropic regulator of carbon flow through glycolysis and other metabolic pathways [70,71]. Interestingly, *fruR *was also shown to regulate the cellular growth and productivity of metabolically-stressed plasmid-bearing cells during fermentation processes [72,73].

### Real-time PCR analysis of stress-related and TCA cycle genes from microarray

A series of additional real-time PCR experiments were carried out to validate the expression of key genes identified from the microarray study (Table 3). Out of ten genes validated, seven are stress-related genes upregulated in the metabolically-stressed scFvD1.3 cells from microarray data, while the other three are down-regulated TCA cycle genes. Although not of the same magnitude, the real-time PCR expression of these genes agreed fairly well with the microarray data. In particular, the real-time PCR expression of six stress-related genes (clpS, dnaJ, fkpB, grpE, hslR, ibpA, ybbN) was consistently higher in absence of chaperone expression, thus validating that these six genes were indeed up-regulated in the metabolically-stressed cells. As possible future work to improve heterologous protein expression, the native promoter sequences of these genes could be cloned upstream of a reporter gene to indicate potential protein misfolding in recombinant cells during fermentation processes.

**Table 3:** Real-time PCR validation of stress-related and TCA cycle genes

	scFvD1.3 vs Skp/scFvD1.3	scFvD1.3 vs FkpA/scFvD1.3
Gene	Real-time PCR expression ratio	
*clpS*	1.6	1.9
*dnaJ*	2.1	3.0
*fkpB*	1.6	1.7
*grpE*	1.8	1.4
*hslR*	2.2	2.5
*ibpA*	-1.1	1.4
*ybbN*	2.5	2.4
*sdhB*	-2.1	-2.7
*sdhD*	-2.5	-2.6
*sucD*	-2.5	-2.4

Quantitative real-time gene expression comparison of the wildtype scFvD1.3 over the chaperone co-expressing Skp/scFvD1.3 or FkpA/scFvD1.3 cells. Seven upregulated stress-related genes and three down-regulated TCA cycle genes from the microarray were validated with real-time PCR.

## Conclusions

The periplasmic expression of scFvD1.3 antibody fragments led to the accumulation of insoluble scFvD1.3 proteins and a decrease in the cell viability of scFvD1.3 cells, therefore providing a model to examine the metabolic stress possibly related to protein misfolding and its associated loss of cell viability. As protein misfolding and aggregation into insoluble inclusion bodies can be prevented via the activity of protein folding factors, three folding factors were co-expressed in parallel with scFvD1.3. It was shown that the co-expression of Skp and FkpA increased scFvD1.3 solubility and cell viability in a dosage-dependent manner. From N-terminal and C-terminal mutation experiments performed on FkpA, it was shown that its chaperone activity is required for the improvement of cell viability. These results further support the notion that protein misfolding is the principal cause of metabolic burden in the scFvD1.3 cells.

Global gene expression analysis of the scFvD1.3 cells over the chaperone-expressing cells showed a clear trend of up-regulation for genes involved in heat-shock response and protein misfolding, including several genes belonging to the major HSP70 DnaK chaperone family and key proteases of the Clp and lon protease systems. Other gene expression trends include: (1) the differential regulation of several energy metabolic genes, (2) down-regulation of the central metabolic TCA cycle and transport genes, and (3) up-regulation of growth-rate dependent ribosomal genes. In all, the parallel transcriptional regulation on metabolic stress related and other genes may constitute the stress response to protein misfolding in the scFvD1.3 cells. These gene expression information could prove to be valuable for the selection and construction of reporter contructs for monitoring of the protein misfolding response during antibody fragment production.

## Methods

### Cloning and bacterial strains

The pET-scFvD1.3 plasmid carrying the *scFvD1.3 *(anti-hen egg lysozyme) gene in an isopropyl β-D-1-thiogalactopyranoside (IPTG)-inducible pET-39b(+) vector (Novagen, USA) was transformed into *Escherichia coli *BL21(DE3) (Invitrogen, USA) to give the scFvD1.3 cells. The *lacZ, dsbA skp *and *fkpA *genes were amplified from *E. coli *genomic DNA and separately cloned into the MCS of the arabinose-inducible pACYCDuet-1 plasmid (Novagen, USA). The plasmids carrying these genes were subsequently transformed into the scFvD1.3 cells to give the: (1) BL21/pET-scFvD1.3/pAra-lacZ, (2) BL21/pET-scFvD1.3/pAra-dsbA, (3) BL21/pET-scFvD1.3/pAra-skp (Skp/scFvD1.3 cells), and (4) BL21/pET-scFvD1.3/pAra-fkpA (FkpA/scFvD1.3 cells) respectively. To examine the effect of co-expressing Skp and FkpA together, both skp & fkpA genes were also cloned in tandem into the MCS of the pACYCDuet-1 vector and transformed into the scFvD1.3 cells.

The N-terminal mutation in FkpA (deletion of amino acid residues 3 to 96) was generated using overlap extension PCR as decribed [15]. The two mega primers were amplified from fkpA using primer N3 (5' ATTCTTTGCCTTTCGCTTCGTTATCCGGTTTCGCCGC TTCCG CCGC 3') and N5 (5' GATAACGAAGCGAAAGGCAAAGAATATC 3') in two separate PCR reactions with the Stratagene pfu turbo mastermix (Stratagene, USA). Thermal cycle conditions were: 95°C for 2 minutes, followed by 35 cycles of 95°C for 30 seconds, 49°C °C for 30 seconds and 72°C for 1 minutes and a final 72°C for 10 minutes. Subsequently, the N3 and N5 products were overlapped by adding 5 microliters of the products in another 25 μl PCR reaction. The reaction was carried out for 10 cycles to generate the template for a third round of PCR using 20 pmoles of flanking primers. For the generation of the FkpA C-terminal mutation, the amino acid substitutions of Ile and Gly at position 174 and 176 to Ser was performed with Quikchange Site-Directed Mutagenesis Kit (Stratagene, CA) according the the manufacturer protocol. A pair of sense (5' CTGGATGGCGTGAGTCCGAGCTGGACCGAAGGC 3') and antisense (5' GCCTTCGGTCCAGCTCGGACTCACGCCATCCAG 3') mutagenesis primers were used and the thermal cycle conditions were: 95°C for 30 seconds, followed by 12 cycles of 95°C for 30 seconds, 55°C for 30 seconds and 68°C for 5 minutes. 10 U of DpnI restriction enzyme was added to each amplification reaction (37°C, 1 hour) before transformation into XL1-Blue supercompetent cells and selection on LB chloramphenicol (34 μg/ml) plates. All mutations of fkpA were confirmed by sequencing before cloning into pACYCDuet-1.

### Shake flask and bioreactor culturing

Kanamycin (50 μg/ml) was used for the selection of the scFvD1.3 cells, while kanamycin (30 μg/ml) and chloramphenicol (34 μg/ml) were used for the selection of chaperone co-expressing cells. Single colonies on LB plates with the appropriate antibiotics were inoculated into 50 mL LB + 0.5% glucose (37°C, 220 rpm) to prepare the starter culture. After 15-18 hours, 5 ml of the the starter culture was inoculated into 50 ml 2×YT media (16 g/L tryphone, 10 g/L yeast extraction, 5 g/L NaCl, 1 g/L glycerol) and cultured at 30°C (220 rpm). When OD600 has reached 0.8-1, IPTG (20 μM) was used for the induction of scFvD1.3 expression and arabinose (0 to 0.2%) was used to co-induce the expression of Skp, FkpA and the other proteins.

20 mL of the starter cultures were inoculated into bioreactors containing 2 L 2×YT media. The following culture parameters were used: 30°C, pH 7.0, minimum 20% dissolved oxygen (DO) cascaded to stirrer (200-800 rpm) followed by airflow (1.5-10 L/min). OD_600 _readings were measured hourly with Libra UV/VIS S22 Spectrophotometer (Biochrom, UK). When OD_600 _reached 4.0-6.0, IPTG (20 μM) and arabinose (0.2%) were added for induction of scFvD1.3 and chaperones (skp and fkpA) respectively. Three hours post-induction, about 5×10^9^-1×10^10 ^bacteria cells were collected and immediately stabilized with 2 volumes of RNAprotect reagent (QIAGEN, Valencia, CA) for 5 min. Subseqently, the samples were centrifuged (4600 rpm, 5 min) and resultant pellets were immediately stored at -80°C for microarray analysis.

### Cell viability and Western blot

Cell samples were collected at alternate hours and diluted with 0.1% peptone (10^3^-10^6 ^dilutions). Samples were plated onto LB and LB+Kan plates and incubated overnight at 37°C for colony counts. 50-100 colonies were picked from LB plates of each time-point with sterile toothpicks, streaked onto LB+Kan and LB plates and incubated overnight at 37°C. Cell viability is taken as the number of colonies on LB+Kan over LB plates. Cell pellets (1 ml) five hours post-induction were resuspended in 500 μl of TBS buffer and subjected to 6 to 8 rounds of sonication on ice using Vibracell Ultrasonic Processor 130 Watt (SONICS), with an interval of 10 seconds. The sonicated samples were then centrifuged at 13,000 rpm for 2 min. The resultant pellet consisted of the cell debris along with the insoluble proteins, while the supernatant consisted of soluble proteins. For SDS-PAGE, the soluble proteins after reduction were loaded onto 4%-12% NuPage polyacrylamide gels (Invitrogen, USA) and ran with a constant voltage of 200 V for 35 mins before coomassie staining. For Western blot, both the soluble and insoluble proteins after SDS-PAGE were transferred to a methanol activated PVDF membrane at constant current of 80 mA per gel for 60 min. After blocking (3% non-fat milk, 20 mM Tris-HCl, pH 7.5 150 mM NaCl, 1% tween 20) for 1 hour with agitation at room temperature, the membrane was washed three times in TBST (20 mM Tris-HCl, pH 7.5 150 mM NaCl, 1% tween 20) and incubated with 1:1000 mouse anti-his-HRP antibody (Merck, Germany) for 2 hours at room temperature. Washing was repeated again three times and the scFvD1.3 proteins visualized with a Bio-Rad alkaline phosphatase conjugate substrate kit as per manufacturer protocol (Bio-Rad, CA, USA).

### Enzyme-linked ImmunoSorbent Assay (ELISA)

Serial dilutions (100 μl) of samples and scFvD1.3 standards were added to microtitre plates (NUNC, Denmark) coated overnight previously with 1 g/L lysozyme solution and incubated at room temperature for 1.5 h. The plates were subsequently washed three times (0.1% Tween-20 in PBS) and incubated with 1:5000 mouse anti-penta-his antibody (QIAGEN, CA, USA) for 1.5 h, followed by washing and a second incubation (1.5 h) with 1:5000 goat anti-mouse IgG horseradish antibody (Sigma, MO, USA). After a final washing step, the plates were incubated in the dark (30 min) with 100 μl TMB substrate solution (Thermo Scientific, MA, USA), before addition of 100 μl 2 M H_2_SO_4 _to halt the reaction. Absorbance values were read by a Sunrise microplate reader (TECAN, Switzerland) at 450ηm with 620ηm reference absorbance.

### Microarray analysis

RNA extraction was performed using QIAGEN RNeasy Extraction Midi Kit (QIAGEN, Valencia, CA) according to manufacturer's instructions. Subsequent procedures were performed according to GeneChip^® ^Expression Analysis Technical Manual (Affymetrix, USA), with reference to the 169 array format of the GeneChip^® ^*E. coli *Genome 2.0 Array. Briefly, cDNA synthesis was carried out by reverse transcription of RNA (~10 μg) using SuperScript II (Invitrogen, Carlsbad, CA) and random primers. cDNA was purified and eluted with MinElute PCR Purification Kit (QIAGEN, Valencia, USA). cDNA was fragmented with 0.6 U/μL DNAseI to produce cDNA fragments between 50-200 bp. cDNA was labeled with GeneChip^® ^Labeling Reagent using terminal deoxynucleotidyl transferase. Labeled cDNA was hybridized to GeneChip^® ^*E. coli *Genome 2.0 Array and incubated in a hybridization oven (45°C, 16 rpm). Array washing and staining was automated by GeneChip^® ^Fluidics Station 450 and scanned with GeneChip^® ^Scanner 3000. Image data was analyzed with GeneChip^® ^Operating Software (GCOS) as described in the GeneChip^® ^expression manual. Data for 4070 *E. coli *K12 probes was exported and analyzed. Comparison expression analysis was performed to compare cell intensities of control probes across different arrays and create a scaling factor for each chip. This quantifies gene expression changes and avoids inaccuracies from probe specific effects. Change algorithms then calculate relative changes in expression levels of transcripts between two separate probe arrays (experiment and baseline) and express them as signal log ratios and change p-values.

## Competing interests

The authors declare that they have no competing interests.

## Authors' contributions

DSO conceived of the research, DSO and VVW participated in its design and coordination; DSO and DYL performed the experiments, interpreted the results and drafted the manuscript; AC and PMN participated in the Western and microarray analysis. All authors read and approved the final manuscript.

## Supplementary Material

Additional file 1 **Up-regulated genes for scFvD.13 cells over the Skp/scFvD1.3 cells**. Fold-change, gene ID and functional information were listed for up-regulated genes from comparison expression analysis of the wildtype scFvD1.3 over the chaperone co-expressing Skp/scFvD1.3 cells. Click here for file

Additional file 2 **Down-regulated genes for scFvD.13 cells over the Skp/scFvD1.3 cells**. Fold-change, gene ID and functional information were listed for down-regulated genes from comparison expression analysis of the wildtype scFvD1.3 over the chaperone co-expressing Skp/scFvD1.3 cells. Click here for file

Additional file 3 **Up-regulated genes for scFvD.13 cells over the FkpA/scFvD1.3 cells**. Fold-change, gene ID and functional information were listed for up-regulated genes from comparison expression analysis of the wildtype scFvD1.3 over the chaperone co-expressing FkpA/scFvD1.3 cells. Click here for file

Additional file 4 **ADown-regulated genes for scFvD.13 cells over the FkpA/scFvD1.3 cells**. Fold-change, gene ID and functional information were listed for down-regulated genes from comparison expression analysis of the wildtype scFvD1.3 over the chaperone co-expressing FkpA/scFvD1.3 cells. Click here for file
