# Supplementary material for: Co-expression of Skp and FkpA chaperones improves cell viability and alters the global expression of stress response genes during scFvD1.3 production
Source: Microb Cell Fact. 2010 Apr 13;9:22. doi: 10.1186/1475-2859-9-22 (PMC2868799; doi:10.1186/1475-2859-9-22)
Supplement: Additional file 1 — Up-regulated genes for scFvD.13 cells over the Skp/scFvD1.3 cells. Fold-change, gene ID and functional information were listed for up-regulated genes from comparison expression analysis of the wildtype scFvD1.3 over the chaperone co-expressing Skp/scFvD1.3 cells. [file 1475-2859-9-22-S1.DOC]

## Additional file 1: Up-regulated genes for scFvD.13 cells over the Skp/scFvD1.3 cells

| **Gene name** | **Probe set name** | **Blattner ID** | **Functional role** | **Fold change** |
| --- | --- | --- | --- | --- |
| *cbl* | 1767711_s_at | b1987 | Transcriptional regulator cbl | 2.2 |
| *clpS* | 1766490_s_at | b0881 | Protein yljA | 3.4 |
| *cydA* | 1763881_s_at | b0733 | Cytochrome D ubiquinol oxidase subunit I (EC 1.10.3.-) | 2.3 |
| *cydB* | 1765588_s_at | b0734 | Cytochrome D ubiquinol oxidase subunit II (EC 1.10.3.-) | 2.1 |
| *cysD* | 1765655_s_at | b2752 | Sulfate adenylyltransferase subunit 2 (EC 2.7.7.4) | 3.1 |
| *cysW* | 1762990_s_at | b2423 | Sulfate transport system permease protein cysW | 1.9 |
| *dnaJ* | 1769019_s_at | b0015 | Chaperone protein dnaJ | 2.2 |
| *gcd* | 1762774_s_at | b0124 | Glucose dehydrogenase [pyrroloquinoline-quinone] (EC 1.1.99.17) | 2.5 |
| *grpE* | 1759164_s_at | b2614 | GrpE protein | 1.9 |
| *hslR* | 1761422_s_at | b3400 | 33 kDa chaperonin | 2.9 |
| *ilvC* | 1767741_at | b3774 | Acetohydroxy acid isomeroreductase | 3.7 |
| *lon* | 1767623_s_at | b0439 | ATP-dependent protease La (EC 3.4.21.53) | 2.4 |
| *menB* | 1765806_s_at | b2262 | Naphthoate synthase (EC 4.1.3.36) | 2.3 |
| *miaA* | 1762453_s_at | b4171 | tRNA delta(2)-isopentenylpyrophosphate transferase (EC 2.5.1.8) | 2.1 |
| *mltD* | 1766197_s_at | b0211 | Membrane-bound lytic murein transglycosylase D precursor (EC 3.2.1.-) | 2.9 |
| *mutM* | 1766244_s_at | b3635 | Formamidopyrimidine-DNA glycosylase (EC 3.2.2.23) | 2.3 |
| *proP* | 1764343_s_at | b4111 | Proline/betaine transporter | 3.5 |
| *ribH* | 1768854_s_at | b0415 | 6,7-dimethyl-8-ribityllumazine synthase (EC 2.5.1.9) | 1.9 |
| *rplR* | 1762255_s_at | b3304 | 50S ribosomal protein L18 | 2.0 |
| *rpmC* | 1762075_s_at | b3312 | 50S ribosomal protein L29 | 1.9 |
| *rpmD* | 1765749_s_at | b3302 | 50S ribosomal protein L30 | 1.9 |
| *rpmI* | 1765538_s_at | b1717 | 50S ribosomal protein L35 | 2.1 |
| *rpsN* | 1763008_s_at | b3307 | 30S ribosomal protein S14 | 2.1 |
| *rpsQ* | 1768397_s_at | b3311 | 30S ribosomal protein S17 | 2.0 |
| *rrmJ* | 1765939_s_at | b3179 | Ribosomal RNA large subunit methyltransferase J (EC 2.1.1.-) | 2.1 |
| *sdaA* | 1767508_s_at | b1814 | L-serine dehydratase 1 (EC 4.2.1.13) | 2.1 |
| *spf* | 1762381_s_at | b3864 | Regulatory-RNAs | 2.3 |
| *tauA* | 1762595_s_at | b0365 | Taurine-binding periplasmic protein precursor | 2.3 |
| *topA* | 1763143_s_at | b1274 | DNA topoisomerase I (EC 5.99.1.2) | 2.4 |
| *ybbN* | 1766513_s_at | b0492 | Hypothetical protein ybbN | 2.1 |
| *yceD* | 1763486_s_at | b1088 | Hypothetical protein yceD | 2.2 |
| *yceP* | 1763516_s_at | b1060 | Hypothetical protein yceP | 2.1 |
| *ycfR* | 1759494_s_at | b1112 | Hypothetical protein ycfR precursor | 5.7 |
| *yciS* | 1762706_s_at | b1279 | Hypothetical protein yciS | 2.1 |
| *ycjF* | 1765041_s_at | b1322 | Hypothetical protein ycjF | 1.9 |
| *ycjX* | 1768270_s_at | b1321 | Hypothetical protein ycjX | 2.2 |
| *ydhQ* | 1764874_s_at | b1664 | Hypothetical protein ydhQ | 2.9 |
| *ydjN* | 1763830_s_at | b1729 | Hypothetical symporter ydjN | 2.5 |
| *yeeD* | 1765487_s_at | b2012 | Hypothetical protein yeeD | 2.1 |
| *yeeE* | 1767951_s_at | b2013 | Hypothetical protein yeeE | 1.9 |
| *yhdN* | 1764710_s_at | b3293 | Hypothetical protein yhdN | 3.7 |
| *yoaE* | 1768340_s_at | b1816 | Hypothetical protein yoaE | 2.0 |
| *yohJ* | 1766731_s_at | b2141 | Hypothetical protein yohJ | 4.6 |
| *zntR* | 1764204_s_at | b3292 | Zn(II)-responsive regulator of zntA | 3.5 |
