# Supplementary material for: Co-expression of Skp and FkpA chaperones improves cell viability and alters the global expression of stress response genes during scFvD1.3 production
Source: Microb Cell Fact. 2010 Apr 13;9:22. doi: 10.1186/1475-2859-9-22 (PMC2868799; doi:10.1186/1475-2859-9-22)
Supplement: Additional file 2 — Down-regulated genes for scFvD.13 cells over the Skp/scFvD1.3 cells. Fold-change, gene ID and functional information were listed for down-regulated genes from comparison expression analysis of the wildtype scFvD1.3 over the chaperone co-expressing Skp/scFvD1.3 cells. [file 1475-2859-9-22-S2.DOC]

## Additional file 2: Down-regulated genes for scFvD.13 cells over the Skp/scFvD1.3 cells

| **Gene name** | **Probe set name** | **Blattner ID** | **Functional role** |  |
| --- | --- | --- | --- | --- |
| *acs* | 1761238_s_at | b4069 | Acetyl-coenzyme A synthetase (EC 6.2.1.1) | **Fold change** |
| *aldA* | 1764023_s_at | b1415 | Aldehyde dehydrogenase A (EC 1.2.1.22) | -4.8 |
| *araC* | 1766543_s_at | b0064 | Arabinose operon regulatory protein | -3.7 |
| *araD* | 1760236_s_at | b0061 | L-ribulose-5-phosphate 4-epimerase (EC 5.1.3.4) | -2.6 |
| *arnT* | 1762877_s_at | b2257 | Hypothetical protein yfbW | -1.6 |
| *artQ* | 1759862_s_at | b0862 | Arginine transport system permease protein artQ | -1.6 |
| *asr* | 1767701_s_at | b1597 | Polypeptide: acid shock protein | -1.6 |
| *astB* | 1760751_s_at | b1745 | Succinylarginine dihydrolase | -4.9 |
| *astC* | 1761406_s_at | b1748 | Succinylornithine transaminase (EC 2.6.1.-) | -3.1 |
| *astD* | 1761460_s_at | b1746 | Succinylglutamic semialdehyde dehydrogenase (EC 1.2.1.-) | -2.6 |
| *astE* | 1760113_s_at | b1744 | Succinylglutamate desuccinylase (EC 3.1.-.-) | -3.1 |
| *betA* | 1762136_at | b0311 | Choline dehydrogenase (EC 1.1.99.1) | -3.6 |
| *carA* | 1759939_s_at | b0032 | Carbamoyl-phosphate synthase small chain (EC 6.3.5.5) | -1.6 |
| *csiD* | 1768235_at | b2659 | Hypothetical protein ygaT | -1.9 |
| *cspD* | 1764295_s_at | b0880 | Cold shock-like protein cspD | -4.1 |
| *cstA* | 1766639_s_at | b0598 | Carbon starvation protein A | -1.7 |
| *cycA* | 1761979_s_at | b4208 | D-serine/D-alanine/glycine transporter | -2.0 |
| *dadX* | 1764811_s_at | b1190 | Alanine racemase, catabolic (EC 5.1.1.1) | -1.9 |
| *ddlB* | 1763831_s_at | b0092 | D-alanine--D-alanine ligase B (EC 6.3.2.4) | -1.7 |
| *dppB* | 1766155_s_at | b3543 | Dipeptide transport system permease protein dppB | -1.9 |
| *dppC* | 1765120_s_at | b3542 | Dipeptide transport system permease protein dppC | -2.1 |
| *envZ* | 1763308_s_at | b3404 | Osmolarity sensor protein envZ (EC 2.7.3.-) | -1.9 |
| *fadA* | 1762184_s_at | b3845 | 3-ketoacyl-CoA thiolase (EC 2.3.1.16) | -1.5 |
| *fadD* | 1764509_s_at | b1805 | Long-chain-fatty-acid--CoA ligase (EC 6.2.1.3) | -4.1 |
| *fadE* | 1761891_at | b0221 | Hypothetical protein yafH | -2.9 |
| *fadH* | 1769205_s_at | b3081 | 2,4-dienoyl-CoA reductase [NADPH] (EC 1.3.1.34) | -2.3 |
| *fadL* | 1760611_s_at | b2344 | Long-chain fatty acid transport protein precursor | -3.0 |
| *flgD* | 1760643_s_at | b1075 | Basal-body rod modification protein flgD | -4.0 |
| *flgG* | 1767435_s_at | b1078 | Flagellar basal-body rod protein flgG | -1.6 |
| *flgH* | 1768045_s_at | b1079 | Flagellar L-ring protein precursor | -1.7 |
| *flgI* | 1765040_s_at | b1080 | Flagellar P-ring protein precursor | -2.2 |
| *flgJ* | 1763207_s_at | b1081 | Peptidoglycan hydrolase flgJ (EC 3.2.1.-) | -2.7 |
| *flgK* | 1761245_s_at | b1082 | Flagellar hook-associated protein 1 | -3.4 |
| *flgL* | 1768710_s_at | b1083 | Flagellar hook-associated protein 3 | -4.8 |
| *flhC* | 1760305_s_at | b1891 | Flagellar transcriptional activator flhC | -2.7 |
| *frdB* | 1765110_s_at | b4153 | Fumarate reductase iron-sulfur protein (EC 1.3.99.1) | -1.5 |
| *frdC* | 1759219_s_at | b4152 | Fumarate reductase 15 kDa hydrophobic protein | -1.7 |
| *ftsI* | 1767626_s_at | b0084 | Peptidoglycan synthetase ftsI precursor | -1.6 |
| *ftsL* | 1766174_s_at | b0083 | Cell division protein ftsL | -3.5 |
| *ftsQ* | 1764724_s_at | b0093 | Cell division protein ftsQ | -2.0 |
| *fucI* | 1769121_s_at | b2802 | L-fucose isomerase (EC 5.3.1.25) | -1.7 |
| *fucK* | 1769242_s_at | b2803 | L-fuculokinase (EC 2.7.1.51) | -4.3 |
| *fucU* | 1763987_s_at | b2804 | Fucose operon fucU protein | -3.9 |
| *gabT* | 1762812_s_at | b2662 | 4-aminobutyrate aminotransferase (EC 2.6.1.19) | -1.7 |
| *galP* | 1760849_s_at | b2943 | Galactose-proton symporter | -2.5 |
| *galS* | 1764520_s_at | b2151 | Mgl repressor and galactose ultrainduction factor | -2.3 |
| *gcvP* | 1765230_s_at | b2903 | Glycine dehydrogenase [decarboxylating] (EC 1.4.4.2) | -2.1 |
| *glcC* | 1767918_s_at | b2980 | Glc operon transcriptional activator | -2.6 |
| *glgS* | 1763125_s_at | b3049 | Glycogen synthesis protein glgS | -2.1 |
| *glgX* | 1759515_s_at | b3431 | Glycogen operon protein glgX (EC 3.2.1.-) | -1.7 |
| *glnG* | 1761372_s_at | b3868 | Nitrogen regulation protein NR(I) | -1.6 |
| *glnH* | 1766180_s_at | b0811 | Glutamine-binding periplasmic protein precursor | -1.7 |
| *glnP* | 1766676_s_at | b0810 | Glutamine transport system permease protein glnP | -1.8 |
| *glnQ* | 1759579_s_at | b0809 | Glutamine transport ATP-binding protein glnQ | -2.2 |
| *gltJ* | 1768428_s_at | b0654 | Glutamate/aspartate transport system permease protein gltJ | -3.0 |
| *gltK* | 1765435_s_at | b0653 | Glutamate/aspartate transport system permease protein gltK | -3.1 |
| *gltL* | 1765895_s_at | b0652 | Glutamate/aspartate transport ATP-binding protein gltL | -2.7 |
| *gntP* | 1768471_s_at | b4321 | High-affinity gluconate transporter | -2.3 |
| *gntT* | 1766810_s_at | b3415 | High-affinity gluconate transporter | -2.1 |
| *gntU* | 1761667_s_at | b3435 | GntU gluconate Gnt transporter | -2.8 |
| *hcaR* | 1766136_s_at | b2537 | Hca operon transcriptional activator | -1.9 |
| *hcaT* | 1768395_s_at | b2536 | Probable 3-phenylpropionic acid transporter | -2.5 |
| *hisA* | 1767282_s_at | b2024 | Phosphoribosylformimino-5-aminoimidazole carboxamide ribonucleotide isomerase | -1.6 |
| *hisM* | 1768853_s_at | b2307 | Histidine transport system permease protein hisM | -1.5 |
| *hisP* | 1765966_s_at | b2306 | Histidine transport ATP-binding protein hisP | -1.6 |
| *hisQ* | 1768624_s_at | b2308 | Histidine transport system permease protein hisQ | -2.3 |
| *leuB* | 1762483_s_at | b0073 | 3-isopropylmalate dehydrogenase (EC 1.1.1.85) | -1.6 |
| *leuD* | 1766336_s_at | b0071 | 3-isopropylmalate dehydratase small subunit (EC 4.2.1.33) | -2.1 |
| *skp* | 1760052_s_at | b0179 | UDP-3-O-[3-hydroxymyristoyl] glucosamine N-acyltransferase (EC 2.3.1.-) | -2.0 |
| *maeB* | 1767376_at | b2463 | NADP-dependent malic enzyme (EC 1.1.1.40) | -3.4 |
| *malT* | 1769204_at | b3418 | MalT transcriptional activator | -1.6 |
| *mazG* | 1767682_s_at | b2781 | MazG protein | -2.1 |
| *mepA* | 1768951_s_at | b2328 | Chorismate synthase (EC 4.6.1.4) | -1.9 |
| *metH* | 1763533_at | b4019 | 5-methyltetrahydrofolate--homocysteine methyltransferase (EC 2.1.1.13) | -1.6 |
| *mglA* | 1762687_s_at | b2149 | Galactoside transport ATP-binding protein mglA | -3.6 |
| *mglB* | 1768444_s_at | b2150 | D-galactose-binding periplasmic protein precursor | -3.0 |
| *mglC* | 1761896_s_at | b2148 | Galactoside transport system permease protein mglC | -1.9 |
| *mgtA* | 1764279_s_at | b4242 | Mg(2+) transport ATPase, P-type 1 (EC 3.6.3.2) | -3.0 |
| *mraW* | 1763957_s_at | b0082 | S-adenosyl-methyltransferase mraW (EC 2.1.1.-) | -2.8 |
| *mraY* | 1759922_s_at | b0087 | Phospho-N-acetylmuramoyl-pentapeptide-transferase (EC 2.7.8.13) | -1.9 |
| *mrdA* | 1761305_s_at | b0635 | Penicillin-binding protein 2 | -1.9 |
| *mtlD* | 1765898_s_at | b3600 | Mannitol-1-phosphate 5-dehydrogenase (EC 1.1.1.17) | -1.6 |
| *mtlR* | 1759389_s_at | b3601 | Mannitol operon repressor | -1.6 |
| *murC* | 1767504_s_at | b0091 | UDP-N-acetylmuramate--alanine ligase (EC 6.3.2.8) | -1.6 |
| *murD* | 1767514_s_at | b0088 | UDP-N-acetylmuramoylalanine--D-glutamate ligase (EC 6.3.2.9) | -2.5 |
| *murG* | 1759861_s_at | b0090 | UDP-N-acetylglucosamine:LPS N-acetylglucosamine transferase (EC 2.4.1.-) | -3.6 |
| *nagE* | 1762936_s_at | b0679 | PTS system, N-acetylglucosamine-specific IIABC component (EC 2.7.1.69) | -2.5 |
| *nanT* | 1759198_s_at | b3224 | Putative sialic acid transporter | -4.0 |
| *ndk* | 1761732_s_at | b2518 | Nucleoside diphosphate kinase (EC 2.7.4.6) | -2.1 |
| *nhaA* | 1767510_s_at | b0019 | Na(+)/H(+) antiporter 1 | -1.7 |
| *nuoA* | 1759438_s_at | b2288 | NADH dehydrogenase I chain A (EC 1.6.5.3) | -2.5 |
| *nuoC* | 1763897_s_at | b2286 | NADH dehydrogenase I chain C/D (EC 1.6.5.3) | -1.7 |
| *nuoE* | 1766657_s_at | b2285 | NADH dehydrogenase I chain E (EC 1.6.5.3) | -2.0 |
| *nuoF* | 1763794_s_at | b2284 | NADH dehydrogenase I chain F (EC 1.6.5.3) | -2.3 |
| *nuoG* | 1766000_s_at | b2283 | NADH dehydrogenase I chain G (EC 1.6.5.3) | -2.1 |
| *nuoH* | 1760147_s_at | b2282 | NADH dehydrogenase I chain H (EC 1.6.5.3) | -4.1 |
| *nuoI* | 1765761_s_at | b2281 | NADH dehydrogenase I chain I (EC 1.6.5.3) | -2.7 |
| *nuoJ* | 1760954_s_at | b2280 | NADH dehydrogenase I chain J (EC 1.6.5.3) | -2.1 |
| *nuoK* | 1766061_s_at | b2279 | NADH dehydrogenase I chain K (EC 1.6.5.3) | -1.9 |
| *nuoL* | 1765545_s_at | b2278 | NADH dehydrogenase I chain L (EC 1.6.5.3) | -2.5 |
| *nuoM* | 1763447_s_at | b2277 | NADH dehydrogenase I chain M (EC 1.6.5.3) | -1.9 |
| *polB* | 1766231_at | b0060 | DNA polymerase II (EC 2.7.7.7) | -3.1 |
| *ppx* | 1768446_s_at | b2502 | Exopolyphosphatase | -2.2 |
| *pstA* | 1766098_s_at | b3726 | Phosphate transport system permease protein pstA | -1.7 |
| *pstB* | 1763997_s_at | b3725 | Phosphate transport ATP-binding protein pstB | -2.3 |
| *pstS* | 1764804_s_at | b3728 | Phosphate-binding periplasmic protein precursor | -2.1 |
| *purB* | 1767455_s_at | b1131 | Adenylosuccinate lyase (EC 4.3.2.2) | -2.3 |
| *purD* | 1769142_at | b4005 | Phosphoribosylamine--glycine ligase (EC 6.3.4.13) | -1.8 |
| *purH* | 1764101_s_at | b4006 | Bifunctional purine biosynthesis protein purH | -2.5 |
| *purL* | 1764318_at | b2557 | Hypothetical protein yfhD | -2.3 |
| *rbsA* | 1760971_s_at | b3749 | Ribose transport ATP-binding protein rbsA | -1.9 |
| *rbsC* | 1763403_s_at | b3750 | Ribose transport system permease protein rbsC | -1.9 |
| *rbsK* | 1763275_s_at | b3752 | Ribokinase (EC 2.7.1.15) | -1.7 |
| *rbsR* | 1767720_s_at | b3753 | RbsR transcriptional repressor | -2.1 |
| *rffA* | 1766616_s_at | b3791 | TDP-4-oxo-6-deoxy-D-glucose transaminase | -1.9 |
| *rffC* | 1762432_s_at | b3790 | Lipopolysaccharide biosynthesis protein rffC | -1.8 |
| *rffH* | 1764595_s_at | b3789 | Glucose-1-phosphate thymidylyltransferase (EC 2.7.7.24) | -1.7 |
| *rrmA* | 1765194_s_at | b1822 | Ribosomal RNA large subunit methyltransferase A (EC 2.1.1.51) | -1.7 |
| *sdaB* | 1767192_s_at | b2797 | L-serine dehydratase 2 (EC 4.2.1.13) | -1.6 |
| *sdaC* | 1768919_s_at | b2796 | Serine transporter | -2.1 |
| *sdhA* | 1762750_s_at | b0723 | Succinate dehydrogenase flavoprotein subunit (EC 1.3.99.1) | -1.7 |
| *sdhB* | 1769260_s_at | b0724 | Succinate dehydrogenase iron-sulfur protein (EC 1.3.99.1) | -1.5 |
| *sdhD* | 1767040_s_at | b0722 | Succinate dehydrogenase hydrophobic membrane anchor protein | -1.9 |
| *soxS* | 1761528_s_at | b4062 | Regulatory protein soxS | -1.9 |
| *spr* | 1764395_s_at | b2175 | Lipoprotein spr precursor | -3.2 |
| *sraH* | 1766206_at | b4450 | Small RNA that interacts with Hfq | -2.1 |
| *sstT* | 1769265_s_at | b3089 | Hypothetical symporter ygjU | -2.1 |
| *sucA* | 1766868_s_at | b0726 | 2-oxoglutarate dehydrogenase E1 component (EC 1.2.4.2) | -2.2 |
| *sucB* | 1760290_s_at | b0727 | Lipoamide acyltransferase subunit of 2-oxoglutarate dehydrogenase complex | -2.9 |
| *sucC* | 1769042_s_at | b0728 | Succinyl-CoA synthetase beta chain (EC 6.2.1.5) | -1.7 |
| *sucD* | 1767719_s_at | b0729 | Succinyl-CoA synthetase alpha chain (EC 6.2.1.5) | -2.8 |
| *tnaA* | 1761050_s_at | b3708 | Tryptophanase (EC 4.1.99.1) | -2.7 |
| *tsx* | 1766260_s_at | b0411 | Nucleoside-specific channel-forming protein tsx precursor | -2.1 |
| *ubiA* | 1765022_s_at | b4040 | 4-hydroxybenzoate octaprenyltransferase (EC 2.5.1.-) | -2.2 |
| *ubiB* | 1762864_s_at | b3835 | Sec-independent protein translocase protein tatA | -1.9 |
| *ubiH* | 1762088_s_at | b2907 | 2-octaprenyl-6-methoxyphenol hydroxylase (EC 1.14.13.-) | -1.9 |
| *uxuB* | 1760272_s_at | b4323 | D-mannonate oxidoreductase (EC 1.1.1.57) | -1.7 |
| *visC* | 1759892_s_at | b2906 | Protein visC | -1.7 |
| *ybhQ* | 1761194_s_at | b0791 | Hypothetical protein ybhQ | -1.9 |
| *ycdN* | 1760869_s_at | b1017 | Pseudogene ycdN | -2.4 |
| *ydhC* | 1766620_s_at | b1660 | Hypothetical transport protein ydhC | -1.8 |
| *yeeI* | 1764330_s_at | b1976 | Hypothetical protein yeeI | -2.1 |
| *yehU* | 1761541_s_at | b2126 | Hypothetical protein yehU precursor | -2.2 |
| *yeiU* | 1761701_s_at | b2174 | Hypothetical protein yeiU | -1.9 |
| *fadJ* | 1761164_s_at | b2341 | Putative fatty oxidation complex alpha subunit | -1.9 |
| *fadI* | 1767812_s_at | b2342 | Probable 3-ketoacyl-CoA thiolase (EC 2.3.1.16) | -3.6 |
| *yhdP* | 1764436_s_at | b3246 | Hypothetical protein yhdP | -3.6 |
| *yhhN* | 1764976_s_at | b3468 | Hypothetical protein yhhN | -1.9 |
| *yjcG* | 1763832_at | b4067 | Putative symporter yjcG | -2.0 |
| *yjcH* | 1767573_s_at | b4068 | Hypothetical protein yjcH | -4.8 |
| *yphF* | 1763073_s_at | b2548 | ABC transporter periplasmic binding protein yphF precursor | -3.1 |
| *yqeF* | 1768275_s_at | b2844 | Probable acetyl-CoA acetyltransferase (EC 2.3.1.9) | -2.2 |
| *yrfF* | 1760252_s_at | b3398 | Putative membrane protein igaA homolog | -2.1 |
| *ytfS* | 1759937_s_at | b4229 | Hypothetical ABC transporter ATP-binding protein ytfR | -1.7 |
