# Supplementary material for: Co-expression of Skp and FkpA chaperones improves cell viability and alters the global expression of stress response genes during scFvD1.3 production
Source: Microb Cell Fact. 2010 Apr 13;9:22. doi: 10.1186/1475-2859-9-22 (PMC2868799; doi:10.1186/1475-2859-9-22)
Supplement: Additional file 3 — Up-regulated genes for scFvD.13 cells over the FkpA/scFvD1.3 cells. Fold-change, gene ID and functional information were listed for up-regulated genes from comparison expression analysis of the wildtype scFvD1.3 over the chaperone co-expressing FkpA/scFvD1.3 cells. [file 1475-2859-9-22-S3.DOC]

## Additional file 3: Up-regulated genes for scFvD.13 cells over the FkpA/scFvD1.3 cells

| **Gene name** | **Probe set name** | **Blattner ID** | **Functional role** | **Fold change** |
| --- | --- | --- | --- | --- |
| *aceE* | 1761664_s_at | b0114 | Pyruvate dehydrogenase E1 component (EC 1.2.4.1) | 3.6 |
| *alr* | 1759988_s_at | b4053 | Aromatic-amino-acid aminotransferase (EC 2.6.1.57) | 1.8 |
| *aqpZ* | 1764805_s_at | b0875 | Aquaporin Z | 1.8 |
| *araB* | 1761659_s_at | b0063 | L-ribulokinase (EC 2.7.1.16) | 2.8 |
| *araD* | 1760236_s_at | b0061 | L-ribulose-5-phosphate 4-epimerase (EC 5.1.3.4) | 3.1 |
| *araE* | 1765707_s_at | b2841 | Arabinose-proton symporter | 4.0 |
| *araJ* | 1764050_s_at | b0396 | Protein araJ precursor | 6.3 |
| *argH* | 1767872_s_at | b3960 | Argininosuccinate lyase (EC 4.3.2.1) | 1.7 |
| *aroF* | 1764254_s_at | b2601 | Phospho-2-dehydro-3-deoxyheptonate aldolase, Tyr-sensitive (EC 4.1.2.15) | 1.9 |
| *aroK* | 1764480_s_at | b3390 | Shikimate kinase I (EC 2.7.1.71) | 2.1 |
| *arsB* | 1760136_s_at | b3502 | Arsenical pump membrane protein | 1.9 |
| *arsR* | 1765593_at | b3501 | Arsenical resistance operon repressor | 3.6 |
| *clpP* | 1763709_s_at | b0437 | ATP-dependent Clp protease proteolytic subunit (EC 3.4.21.92) | 2.2 |
| *clpS* | 1766490_s_at | b0881 | Protein yljA | 3.1 |
| *clpX* | 1762543_s_at | b0438 | ATP-dependent Clp protease ATP-binding subunit clpX | 2.5 |
| *cmk* | 1762437_s_at | b0910 | Cytidylate kinase (EC 2.7.4.14) | 2.8 |
| *cobS* | 1761590_s_at | b1992 | Cobalamin [5'-phosphate] synthase | 1.7 |
| *csdA* | 1764635_s_at | b2810 | Cysteine sulfinate desulfinase (EC 4.4.1.-) | 1.7 |
| *cspA* | 1761114_s_at | b3556 | Cold shock protein cspA | 2.4 |
| *cutE* | 1767311_s_at | b0657 | Apolipoprotein N-acyltransferase (EC 2.3.1.-) | 4.9 |
| *cyaA* | 1759548_s_at | b3806 | Porphobilinogen deaminase (EC 4.3.1.8) | 2.1 |
| *cysD* | 1765655_s_at | b2752 | Sulfate adenylyltransferase subunit 2 (EC 2.7.7.4) | 7.0 |
| *cysH* | 1759826_s_at | b2762 | Phosphoadenosine phosphosulfate reductase (EC 1.8.99.4) | 2.7 |
| *cysJ* | 1765526_s_at | b2764 | Sulfite reductase [NADPH] flavoprotein alpha-component (EC 1.8.1.2) | 6.3 |
| *cysK* | 1762560_s_at | b2414 | Cysteine synthase A (EC 4.2.99.8) | 2.0 |
| *cysM* | 1761178_s_at | b2421 | Cysteine synthase B (EC 4.2.99.8) | 4.3 |
| *cysW* | 1762990_s_at | b2423 | Sulfate transport system permease protein cysW | 5.5 |
| *deaD* | 1766069_s_at | b3162 | Cold-shock DEAD-box protein A | 4.4 |
| *der* | 1767500_s_at | b2511 | Probable GTP-binding protein engA | 1.9 |
| *dnaG* | 1764525_s_at | b3066 | DNA primase (EC 2.7.7.-) | 1.7 |
| *dnaJ* | 1769019_s_at | b0015 | Chaperone protein dnaJ | 3.1 |
| *dsbC* | 1764795_s_at | b2893 | Thiol:disulfide interchange protein dsbC precursor | 2.1 |
| *dusB* | 1764702_s_at | b3260 | Hypothetical protein yhdG | 3.9 |
| *ecfK* | 1766558_s_at | b0177 | Unknown protein from 2D-page spots M62/M63/O3/O9/T35 precursor | 2.3 |
| *emrD* | 1761644_s_at | b3673 | Multidrug resistance protein D | 2.4 |
| *emrR* | 1766481_s_at | b2684 | Transcriptional repressor mprA | 3.6 |
| *fabD* | 1762314_s_at | b1092 | Malonyl CoA-acyl carrier protein transacylase (EC 2.3.1.39) | 1.9 |
| *fis* | 1767674_s_at | b3261 | DNA-binding protein fis | 3.6 |
| *fkpB* | 1763018_s_at | b0028 | FKBP-type 16 kDa peptidyl-prolyl cis-trans isomerase (EC 5.2.1.8) | 3.7 |
| *flgC* | 1768120_s_at | b1074 | Flagellar basal-body rod protein flgC | 3.0 |
| *flgG* | 1767435_s_at | b1078 | Flagellar basal-body rod protein flgG | 2.8 |
| *flgM* | 1768555_s_at | b1071 | Negative regulator of flagellin synthesis | 2.2 |
| *folD* | 1766006_s_at | b0529 | FolD bifunctional protein | 1.7 |
| *fusA* | 1761999_s_at | b3340 | Elongation factor G | 2.9 |
| *gcd* | 1762774_s_at | b0124 | Glucose dehydrogenase [pyrroloquinoline-quinone] (EC 1.1.99.17) | 2.8 |
| *glmM* | 1766327_at | b3176 | Protein mrsA | 2.2 |
| *glnS* | 1765309_s_at | b0680 | Glutaminyl-tRNA synthetase (EC 6.1.1.18) | 1.8 |
| *glpE* | 1765842_s_at | b3425 | Thiosulfate sulfurtransferase glpE (EC 2.8.1.1) | 1.9 |
| *gntY* | 1760796_s_at | b3414 | Protein yhgI | 2.3 |
| *gpp* | 1764195_s_at | b3779 | Guanosine-5'-triphosphate,3'-diphosphate pyrophosphatase (EC 3.6.1.40) | 2.2 |
| *grpE* | 1759164_s_at | b2614 | GrpE protein | 2.4 |
| *gsk* | 1763194_s_at | b0477 | Inosine-guanosine kinase (EC 2.7.1.73) | 1.8 |
| *guaB* | 1760500_at | b2508 | Inosine-5'-monophosphate dehydrogenase (EC 1.1.1.205) | 2.4 |
| *gyrB* | 1759510_s_at | b3699 | DNA gyrase subunit B (EC 5.99.1.3) | 2.5 |
| *hepA* | 1767289_s_at | b0059 | RNA polymerase associated protein | 2.5 |
| *hflB* | 1769273_s_at | b3178 | Cell division protein ftsH (EC 3.4.24.-) | 3.5 |
| *hflC* | 1761248_s_at | b4175 | HflC protein | 3.4 |
| *hflK* | 1760205_s_at | b4174 | HflK protein | 4.1 |
| *hflX* | 1765516_s_at | b4173 | GTP-binding protein hflX | 2.2 |
| *hisS* | 1767493_s_at | b2514 | Histidyl-tRNA synthetase (EC 6.1.1.21) | 1.9 |
| *holC* | 1762551_s_at | b4259 | DNA polymerase III, chi subunit (EC 2.7.7.7) | 4.6 |
| *hslO* | 1761806_s_at | b3401 | Hypothetical protein yhgE | 5.1 |
| *hslR* | 1761422_s_at | b3400 | 33 kDa chaperonin | 4.9 |
| *hslU* | 1762240_s_at | b3931 | ATP-dependent hsl protease ATP-binding subunit hslU | 2.0 |
| *hslV* | 1766949_s_at | b3932 | ATP-dependent protease hslV (EC 3.4.25.-) | 2.1 |
| *hspQ* | 1765036_s_at | b0966 | Hypothetical protein yccV | 2.1 |
| *htpG* | 1761819_s_at | b0473 | Chaperone protein htpG | 2.5 |
| *htpX* | 1760965_s_at | b1829 | Probable protease htpX (EC 3.4.24.-) | 2.5 |
| *iaaA* | 1766252_s_at | b0828 | Putative L-asparaginase precursor (EC 3.5.1.1) | 2.4 |
| *ibpA* | 1769060_s_at | b3687 | 16 kDa heat shock protein A | 1.9 |
| *ileS* | 1761752_s_at | b0026 | Isoleucyl-tRNA synthetase (EC 6.1.1.5) | 4.3 |
| *ilvC* | 1767741_at | b3774 | Acetohydroxy acid isomeroreductase | 5.1 |
| *infB* | 1767731_s_at | b3168 | Translation initiation factor IF-2 | 3.1 |
| *ldhA* | 1765416_s_at | b1380 | D-lactate dehydrogenase (EC 1.1.1.28) | 2.4 |
| *leuS* | 1763031_s_at | b0642 | Leucyl-tRNA synthetase (EC 6.1.1.4) | 1.7 |
| *lolC* | 1760814_s_at | b1116 | Lipoprotein releasing system transmembrane protein lolC | 1.6 |
| *lon* | 1767623_s_at | b0439 | ATP-dependent protease La (EC 3.4.21.53) | 2.7 |
| *lpxP* | 1759732_s_at | b2378 | DDG protein | 2.6 |
| *lspA* | 1759196_s_at | b0027 | Lipoprotein signal peptidase (EC 3.4.23.36) | 3.0 |
| *mdtD* | 1764160_s_at | b2077 | Hypothetical transport protein yegB | 2.8 |
| *mdtH* | 1769054_s_at | b1065 | Hypothetical transport protein yceL | 2.3 |
| *menB* | 1765806_s_at | b2262 | Naphthoate synthase (EC 4.1.3.36) | 3.2 |
| *menC* | 1759313_s_at | b2261 | O-succinylbenzoate-CoA synthase (EC 4.2.1.-) | 2.8 |
| *mfd* | 1768082_s_at | b1114 | Transcription-repair coupling factor | 3.0 |
| *miaA* | 1762453_s_at | b4171 | tRNA delta(2)-isopentenylpyrophosphate transferase (EC 2.5.1.8) | 2.2 |
| *mltD* | 1766197_s_at | b0211 | Membrane-bound lytic murein transglycosylase D precursor (EC 3.2.1.-) | 4.8 |
| *mrcA* | 1768022_s_at | b3396 | Penicillin-binding protein 1A | 2.1 |
| *mrdA* | 1761305_s_at | b0635 | Penicillin-binding protein 2 | 2.0 |
| *msbA* | 1768530_s_at | b0914 | Probable transport ATP-binding protein msbA | 2.5 |
| *mscS* | 1762370_s_at | b2924 | Hypothetical protein yggB | 1.9 |
| *mutM* | 1766244_s_at | b3635 | Formamidopyrimidine-DNA glycosylase (EC 3.2.2.23) | 3.6 |
| *narP* | 1766995_s_at | b2193 | Nitrate/nitrite response regulator protein narP | 2.2 |
| *nepI* | 1763078_at | b3662 | Hypothetical protein yicM | 1.9 |
| *nusA* | 1762748_s_at | b3169 | N utilization substance protein A | 2.4 |
| *obgE* | 1760326_s_at | b3183 | Hypothetical GTP-binding protein yhbZ | 4.3 |
| *pheS* | 1766499_s_at | b1714 | Phenylalanyl-tRNA synthetase alpha chain (EC 6.1.1.20) | 2.6 |
| *pheT* | 1761950_s_at | b1713 | Phenylalanyl-tRNA synthetase beta chain (EC 6.1.1.20) | 2.4 |
| *potA* | 1759599_s_at | b1126 | Spermidine/putrescine transport ATP-binding protein potA | 1.7 |
| *ppiC* | 1765631_s_at | b3775 | Peptidyl-prolyl cis-trans isomerase C (EC 5.2.1.8) | 1.9 |
| *prc* | 1764737_s_at | b1830 | Tail-specific protease precursor (EC 3.4.21.-) | 1.7 |
| *prfB* | 1768494_s_at | b2891 | Peptide chain release factor 2 | 1.9 |
| *prfC* | 1765689_s_at | b4375 | Peptide chain release factor 3 | 2.4 |
| *priB* | 1766425_s_at | b4201 | Primosomal replication protein N | 3.5 |
| *prlC* | 1761621_s_at | b3498 | Oligopeptidase A (EC 3.4.24.70) | 2.5 |
| *proP* | 1764343_s_at | b4111 | Proline/betaine transporter | 6.3 |
| *proW* | 1762777_s_at | b2678 | Glycine betaine/L-proline transport system permease protein proW | 3.5 |
| *psiE* | 1769010_s_at | b4030 | Hypothetical protein yjbA | 2.4 |
| *queA* | 1768690_s_at | b0405 | S-adenosylmethionine:tRNA ribosyltransferase-isomerase | 1.9 |
| *rbfA* | 1761845_s_at | b3167 | Ribosome-binding factor A | 3.1 |
| *rdgB* | 1763607_s_at | b2954 | Hypothetical protein yggV | 1.9 |
| *recF* | 1764113_s_at | b3700 | DNA replication and repair protein recF | 3.1 |
| *ribH* | 1768854_s_at | b0415 | 6,7-dimethyl-8-ribityllumazine synthase (EC 2.5.1.9) | 2.6 |
| *rimM* | 1767883_s_at | b2608 | 16S rRNA processing protein rimM | 3.1 |
| *rlpB* | 1766757_s_at | b0641 | Rare lipoprotein B precursor | 2.4 |
| *rluA* | 1764559_s_at | b0058 | Ribosomal large subunit pseudouridine synthase A (EC 4.2.1.70) | 3.5 |
| *rluB* | 1761441_s_at | b1269 | Hypothetical protein yciL | 2.7 |
| *rnb* | 1761544_s_at | b1286 | Exoribonuclease II (EC 3.1.13.1) | 2.3 |
| *rnc* | 1759102_s_at | b2567 | Ribonuclease III (EC 3.1.26.3) | 2.5 |
| *rng* | 1760498_s_at | b3247 | Ribonuclease G (EC 3.1.4.-) | 2.0 |
| *rnhB* | 1765273_s_at | b0183 | Ribonuclease HII (EC 3.1.26.4) | 2.0 |
| *rnxG* | 1762187_s_at | b1631 | Electron transport complex protein rnfG | 2.2 |
| *rplA* | 1762846_s_at | b3984 | 50S ribosomal protein L1 | 2.6 |
| *rplB* | 1760752_s_at | b3317 | 50S ribosomal protein L2 | 4.8 |
| *rplC* | 1765697_s_at | b3320 | 50S ribosomal protein L3 | 3.7 |
| *rplD* | 1761917_s_at | b3319 | 50S ribosomal protein L4 | 3.5 |
| *rplE* | 1767821_s_at | b3308 | 50S ribosomal protein L5 | 2.5 |
| *rplF* | 1766400_s_at | b3305 | 50S ribosomal protein L6 | 3.7 |
| *rplI* | 1765561_s_at | b4203 | 50S ribosomal protein L9 | 3.7 |
| *rplJ* | 1759694_s_at | b3985 | 50S ribosomal protein L10 | 2.1 |
| *rplK* | 1761300_s_at | b3983 | 50S ribosomal protein L11 | 2.7 |
| *rplL* | 1761872_s_at | b3986 | 50S ribosomal protein L7/L12 | 2.1 |
| *rplM* | 1762214_s_at | b3231 | 50S ribosomal protein L13 | 2.9 |
| *rplO* | 1764794_s_at | b3301 | 50S ribosomal protein L15 | 3.1 |
| *rplP* | 1759352_s_at | b3313 | 50S ribosomal protein L16 | 3.9 |
| *rplQ* | 1765986_s_at | b3294 | 50S ribosomal protein L17 | 3.4 |
| *rplR* | 1762255_s_at | b3304 | 50S ribosomal protein L18 | 3.9 |
| *rplS* | 1767377_s_at | b2606 | 50S ribosomal protein L19 | 2.8 |
| *rplT* | 1763638_s_at | b1716 | 50S ribosomal protein L20 | 2.6 |
| *rplU* | 1760646_s_at | b3186 | 50S ribosomal protein L21 | 1.9 |
| *rplV* | 1761244_s_at | b3315 | 50S ribosomal protein L22 | 3.7 |
| *rplW* | 1762454_s_at | b3318 | 50S ribosomal protein L23 | 4.1 |
| *rplX* | 1767321_s_at | b3309 | 50S ribosomal protein L24 | 2.2 |
| *rplY* | 1765968_s_at | b2185 | 50S ribosomal protein L25 | 2.3 |
| *rpmA* | 1766441_s_at | b3185 | 50S ribosomal protein L27 | 2.1 |
| *rpmB* | 1762334_s_at | b3637 | 50S ribosomal protein L28 | 2.1 |
| *rpmC* | 1762075_s_at | b3312 | 50S ribosomal protein L29 | 5.1 |
| *rpmD* | 1765749_s_at | b3302 | 50S ribosomal protein L30 | 3.9 |
| *rpmE* | 1762036_s_at | b3936 | 50S ribosomal protein L31 | 2.6 |
| *rpmI* | 1765538_s_at | b1717 | 50S ribosomal protein L35 | 2.6 |
| *rpmJ* | 1768595_s_at | b3299 | 50S ribosomal protein L36 | 2.7 |
| *rpoA* | 1767563_s_at | b3295 | DNA-directed RNA polymerase alpha chain (EC 2.7.7.6) | 2.7 |
| *rpoB* | 1760225_s_at | b3987 | DNA-directed RNA polymerase beta chain (EC 2.7.7.6) | 3.6 |
| *rpoC* | 1764311_s_at | b3988 | DNA-directed RNA polymerase beta' chain (EC 2.7.7.6) | 3.6 |
| *rpoD* | 1765431_s_at | b3067 | RNA polymerase sigma factor rpoD | 4.6 |
| *rpsA* | 1762512_s_at | b0911 | 30S ribosomal protein S1 | 3.0 |
| *rpsB* | 1768873_s_at | b0169 | 30S ribosomal protein S2 | 2.2 |
| *rpsC* | 1765067_s_at | b3314 | 30S ribosomal protein S3 | 4.9 |
| *rpsD* | 1765207_s_at | b3296 | 30S ribosomal protein S4 | 2.8 |
| *rpsE* | 1765151_s_at | b3303 | 30S ribosomal protein S5 | 3.5 |
| *rpsG* | 1762933_s_at | b3341 | 30S ribosomal protein S7 | 2.9 |
| *rpsH* | 1768794_s_at | b3306 | 30S ribosomal protein S8 | 3.2 |
| *rpsI* | 1768660_s_at | b3230 | 30S ribosomal protein S9 | 2.8 |
| *rpsJ* | 1767291_s_at | b3321 | 30S ribosomal protein S10 | 3.6 |
| *rpsK* | 1762768_s_at | b3297 | 30S ribosomal protein S11 | 2.9 |
| *rpsM* | 1768867_s_at | b3298 | 30S ribosomal protein S13 | 2.5 |
| *rpsN* | 1763008_s_at | b3307 | 30S ribosomal protein S14 | 3.0 |
| *rpsP* | 1765175_s_at | b2609 | 30S ribosomal protein S16 | 2.9 |
| *rpsQ* | 1768397_s_at | b3311 | 30S ribosomal protein S17 | 3.7 |
| *rpsR* | 1762305_s_at | b4202 | 30S ribosomal protein S18 | 4.0 |
| *rpsS* | 1761856_s_at | b3316 | 30S ribosomal protein S19 | 3.7 |
| *rrmJ* | 1765939_s_at | b3179 | Ribosomal RNA large subunit methyltransferase J (EC 2.1.1.-) | 2.8 |
| *sdaA* | 1767508_s_at | b1814 | L-serine dehydratase 1 (EC 4.2.1.13) | 3.4 |
| *secA* | 1764003_s_at | b0098 | Preprotein translocase secA subunit | 2.0 |
| *secY* | 1767404_s_at | b3300 | Preprotein translocase secY subunit | 2.8 |
| *sotB* | 1760766_s_at | b1528 | Sugar efflux transporter | 2.2 |
| *spf* | 1762381_s_at | b3864 | Regulatory-RNAs | 2.5 |
| *srmB* | 1761737_at | b2576 | ATP-dependent RNA helicase srmB | 2.3 |
| *tatB* | 1766783_s_at | b3838 | Component of the TatABCE (twin-arginine translocation) complex | 2.1 |
| *tauA* | 1762595_s_at | b0365 | Taurine-binding periplasmic protein precursor | 5.7 |
| *thrA* | 1759122_s_at | b0002 | Bifunctional aspartokinase/homoserine dehydrogenase I | 2.8 |
| *thrB* | 1765942_s_at | b0003 | Homoserine kinase (EC 2.7.1.39) | 2.5 |
| *thrC* | 1761169_s_at | b0004 | Threonine synthase (EC 4.2.3.1) | 1.7 |
| *tolA* | 1766262_s_at | b0739 | TolA protein | 2.5 |
| *tolR* | 1767600_s_at | b0738 | TolR protein | 2.6 |
| *topA* | 1763143_s_at | b1274 | DNA topoisomerase I (EC 5.99.1.2) | 3.5 |
| *trmD* | 1764493_s_at | b2607 | tRNA (Guanine-N(1)-)-methyltransferase (EC 2.1.1.31) | 3.1 |
| *truB* | 1766161_s_at | b3166 | tRNA pseudouridine synthase B (EC 4.2.1.70) | 2.6 |
| *typA* | 1767503_s_at | b3871 | GTP-binding protein typA/BipA | 3.4 |
| *valS* | 1766346_s_at | b4258 | Valyl-tRNA synthetase (EC 6.1.1.9) | 4.6 |
| *xerD* | 1765266_s_at | b2894 | Integrase/recombinase xerD | 2.5 |
| *yaeL* | 1768561_s_at | b0176 | Protease ecfE (EC 3.4.24.-) | 3.4 |
| *yafD* | 1767267_s_at | b0209 | Hypothetical protein yafD | 2.0 |
| *ybbN* | 1766513_s_at | b0492 | Hypothetical protein ybbN | 3.5 |
| *ybeA* | 1766638_s_at | b0636 | Hypothetical protein ybeA | 1.7 |
| *ybeD* | 1766049_s_at | b0631 | Hypothetical protein ybeD | 3.1 |
| *ybeX* | 1761804_s_at | b0658 | Magnesium and cobalt efflux protein corC | 3.2 |
| *ybeY* | 1759770_s_at | b0659 | Hypothetical protein ybeY | 2.8 |
| *ybeZ* | 1759574_s_at | b0660 | PhoH-like protein | 2.2 |
| *ybjX* | 1760844_s_at | b0877 | Hypothetical protein ybjX | 2.0 |
| *yceD* | 1763486_s_at | b1088 | Hypothetical protein yceD | 3.1 |
| *yceP* | 1763516_s_at | b1060 | Hypothetical protein yceP | 2.3 |
| *ycfR* | 1759494_s_at | b1112 | Hypothetical protein ycfR precursor | 4.9 |
| *yciM* | 1768989_s_at | b1280 | Hypothetical protein yciM precursor | 4.8 |
| *yciS* | 1762706_s_at | b1279 | Hypothetical protein yciS | 2.1 |
| *ycjF* | 1765041_s_at | b1322 | Hypothetical protein ycjF | 4.4 |
| *ycjX* | 1768270_s_at | b1321 | Hypothetical protein ycjX | 4.0 |
| *ydhQ* | 1764874_s_at | b1664 | Hypothetical protein ydhQ | 3.5 |
| *ydjN* | 1763830_s_at | b1729 | Hypothetical symporter ydjN | 7.0 |
| *yebC* | 1760813_s_at | b1864 | Protein yebC | 1.7 |
| *yeeD* | 1765487_s_at | b2012 | Hypothetical protein yeeD | 5.5 |
| *yeeE* | 1767951_s_at | b2013 | Hypothetical protein yeeE | 4.8 |
| *yfcJ* | 1765569_s_at | b2322 | Hypothetical protein yfcJ | 4.0 |
| *yfgB* | 1767666_s_at | b2517 | Hypothetical protein yfgB | 1.8 |
| *yfgM* | 1764704_s_at | b2513 | Hypothetical protein yfgM | 1.8 |
| *ygeA* | 1762627_s_at | b2840 | Hypothetical protein ygeA | 4.0 |
| *ygeD* | 1766090_s_at | b2835 | Hypothetical protein ygeD | 2.1 |
| *ygiQ* | 1764572_s_at | b3015 | Polypeptide: conserved protein | 2.5 |
| *yhcN* | 1759182_s_at | b3238 | Hypothetical protein yhcN precursor | 2.9 |
| *yhdN* | 1764710_s_at | b3293 | Hypothetical protein yhdN | 4.6 |
| *yhhS* | 1766580_s_at | b3473 | Hypothetical protein yhhS | 3.1 |
| *yjgP* | 1768492_s_at | b4261 | Hypothetical protein yjgP | 3.0 |
| *yncJ* | 1763859_s_at | b1436 | Hypothetical protein yncJ precursor | 2.0 |
| *yohJ* | 1766731_s_at | b2141 | Hypothetical protein yohJ | 4.3 |
| *yrbD* | 1767705_s_at | b3193 | Hypothetical protein yrbD precursor | 1.9 |
| *zntR* | 1764204_s_at | b3292 | Zn(II)-responsive regulator of zntA | 4.0 |
