# Supplementary material for: Co-expression of Skp and FkpA chaperones improves cell viability and alters the global expression of stress response genes during scFvD1.3 production
Source: Microb Cell Fact. 2010 Apr 13;9:22. doi: 10.1186/1475-2859-9-22 (PMC2868799; doi:10.1186/1475-2859-9-22)
Supplement: Additional file 4 — ADown-regulated genes for scFvD.13 cells over the FkpA/scFvD1.3 cells. Fold-change, gene ID and functional information were listed for down-regulated genes from comparison expression analysis of the wildtype scFvD1.3 over the chaperone co-expressing FkpA/scFvD1.3 cells. [file 1475-2859-9-22-S4.DOC]

## Additional file 4: Down-regulated genes for scFvD.13 cells over the FkpA/scFvD1.3 cells

| **Gene name** | **Probe Set Name** | **Blattner ID** | **Functional role** | **Fold change** |
| --- | --- | --- | --- | --- |
| *abgA* | 1761838_s_at | b1338 | Aminobenzoyl-glutamate utilization protein A | -4.6 |
| *aceA* | 1763981_s_at | b4015 | Isocitrate lyase (EC 4.1.3.1) | -4.6 |
| *aceB* | 1762343_s_at | b4014 | Malate synthase A (EC 4.1.3.2) | -2.5 |
| *aceK* | 1762781_at | b4016 | Isocitrate dehydrogenase kinase/phosphatase (EC 2.7.1.116) (EC 3.1.3.-) | -1.7 |
| *acnA* | 1760881_s_at | b1276 | Aconitate hydratase 1 (EC 4.2.1.3) | -6.7 |
| *acs* | 1761238_s_at | b4069 | Acetyl-coenzyme A synthetase (EC 6.2.1.1) | -6.7 |
| *aldA* | 1764023_s_at | b1415 | Aldehyde dehydrogenase A (EC 1.2.1.22) | -3.1 |
| *aldB* | 1765157_s_at | b3588 | Aldehyde dehydrogenase B (EC 1.2.1.22) | -2.9 |
| *allA* | 1760508_s_at | b0505 | Ureidoglycolate hydrolase (EC 3.5.3.19) | -2.5 |
| *anmK* | 1760946_s_at | b1640 | Hypothetical protein ydhH | -1.7 |
| *aphA* | 1759927_s_at | b4055 | Class B acid phosphatase precursor (EC 3.1.3.2) | -1.9 |
| *argT* | 1766693_at | b2310 | Lysine-arginine-ornithine-binding periplasmic protein precursor | -3.1 |
| *astB* | 1760751_s_at | b1745 | Succinylarginine dihydrolase | -6.5 |
| *astC* | 1761406_s_at | b1748 | Succinylornithine transaminase (EC 2.6.1.-) | -5.3 |
| *astD* | 1761460_s_at | b1746 | Succinylglutamic semialdehyde dehydrogenase (EC 1.2.1.-) | -5.7 |
| *astE* | 1760113_s_at | b1744 | Succinylglutamate desuccinylase | -7.2 |
| *atoD* | 1768251_s_at | b2221 | Acetate CoA-transferase alpha subunit (EC 2.8.3.8) | -2.6 |
| *bfr* | 1764371_s_at | b3336 | Bacterioferritin | -3.7 |
| *blc* | 1766770_s_at | b4149 | Beta-lactamase precursor (EC 3.5.2.6) | -2.1 |
| *bolA* | 1760874_s_at | b0435 | BolA protein | -2.6 |
| *chbB* | 1764757_s_at | b1738 | PTS system, cellobiose-specific IIB component (EC 2.7.1.69) | -1.9 |
| *csiD* | 1768235_at | b2659 | Hypothetical protein ygaT | -5.7 |
| *csiE* | 1761243_s_at | b2535 | Stationary phase inducible protein csiE | -2.9 |
| *cspD* | 1764295_s_at | b0880 | Cold shock-like protein cspD | -3.5 |
| *cspE* | 1766690_s_at | b0623 | Cold shock-like protein cspE | -2.9 |
| *cstA* | 1766639_s_at | b0598 | Carbon starvation protein A | -2.0 |
| *cyaR* | 1763679_at | b4438 | Regulatory-RNAs | -3.1 |
| *cycA* | 1761979_s_at | b4208 | D-serine/D-alanine/glycine transporter | -2.4 |
| *dadA* | 1759906_s_at | b1189 | D-amino acid dehydrogenase small subunit (EC 1.4.99.1) | -1.9 |
| *dadX* | 1764811_s_at | b1190 | Alanine racemase, catabolic (EC 5.1.1.1) | -2.5 |
| *ddpX* | 1764014_s_at | b1488 | D-alanyl-D-alanine dipeptidase (EC 3.4.13.-) | -2.6 |
| *dkgA* | 1764019_s_at | b3012 | 2,5-diketo-D-gluconic acid reductase A (EC 1.1.1.-) | -1.9 |
| *dppA* | 1759395_s_at | b3544 | Periplasmic dipeptide transport protein precursor | -2.7 |
| *dps* | 1759309_s_at | b0812 | DNA protection during starvation protein | -2.6 |
| *ecfJ* | 1767679_s_at | b4216 | Protein ytfJ precursor | -2.8 |
| *ecnB* | 1766745_s_at | b4411 | EcnAB regulate programmed bacterial cell death under high osmolarity conditions | -5.1 |
| *elaB* | 1760724_s_at | b2266 | ElaB protein | -2.3 |
| *erfK* | 1766241_at | b1990 | Protein erfK/srfK precursor | -1.7 |
| *fadA* | 1762184_s_at | b3845 | 3-ketoacyl-CoA thiolase (EC 2.3.1.16) | -5.3 |
| *fadD* | 1764509_s_at | b1805 | Long-chain-fatty-acid--CoA ligase (EC 6.2.1.3) | -4.0 |
| *fadE* | 1761891_at | b0221 | Hypothetical protein yafH | -3.5 |
| *fadH* | 1769205_s_at | b3081 | 2,4-dienoyl-CoA reductase [NADPH] (EC 1.3.1.34) | -4.9 |
| *fadI* | 1767812_s_at | b2342 | Probable 3-ketoacyl-CoA thiolase (EC 2.3.1.16) | -4.4 |
| *fadJ* | 1761164_s_at | b2341 | Putative fatty oxidation complex alpha subunit | -3.9 |
| *fadL* | 1760611_s_at | b2344 | Long-chain fatty acid transport protein precursor | -6.3 |
| *fbaB* | 1759621_s_at | b2097 | Class I fructose 1,6 bisphosphate aldolases | -3.2 |
| *feaR* | 1768900_at | b1384 | Transcriptional activator feaR | -2.9 |
| *fkpA* | 1759106_s_at | b3347 | FKBP-type peptidyl-prolyl cis-trans isomerase fkpA precursor (EC 5.2.1.8) | -10.9 |
| *frdB* | 1765110_s_at | b4153 | Fumarate reductase iron-sulfur protein (EC 1.3.99.1) | -1.8 |
| *fruR* | 1760828_s_at | b0080 | Fructose repressor | -1.7 |
| *fucI* | 1769121_s_at | b2802 | L-fucose isomerase (EC 5.3.1.25) | -2.2 |
| *fucR* | 1768843_s_at | b2805 | L-fucose operon activator | -2.5 |
| *fumA* | 1767184_s_at | b1612 | Fumarate hydratase class I, aerobic (EC 4.2.1.2) | -2.1 |
| *fumC* | 1768359_s_at | b1611 | Fumarate hydratase class II (EC 4.2.1.2) | -1.6 |
| *gabD* | 1760046_s_at | b2661 | Succinate-semialdehyde dehydrogenase [NADP+] (EC 1.2.1.16) | -7.0 |
| *gabP* | 1764229_s_at | b2663 | GABA permease | -2.8 |
| *gabT* | 1762812_s_at | b2662 | 4-aminobutyrate aminotransferase (EC 2.6.1.19) | -4.0 |
| *galP* | 1760849_s_at | b2943 | Galactose-proton symporter | -4.1 |
| *galS* | 1764520_s_at | b2151 | Mgl repressor and galactose ultrainduction factor | -3.6 |
| *gatY* | 1767672_s_at | b2096 | Tagatose-bisphosphate aldolase gatY (EC 4.1.2.-) | -1.7 |
| *gatZ* | 1762947_s_at | b2095 | Putative tagatose 6-phosphate kinase gatZ (EC 2.7.1.144) | -2.0 |
| *glcA* | 1759891_s_at | b2975 | Glycolate permease glcA | -3.2 |
| *glcB* | 1764114_at | b2976 | Malate synthase G | -5.5 |
| *glcC* | 1767918_s_at | b2980 | Glc operon transcriptional activator | -2.1 |
| *glcD* | 1767000_s_at | b2979 | Glycolate oxidase subunit glcD | -6.3 |
| *glcF* | 1764947_at | b2978 | Glycolate oxidase iron-sulfur subunit | -3.5 |
| *glcG* | 1765708_s_at | b2977 | Protein glcG | -10.6 |
| *glgS* | 1763125_s_at | b3049 | Glycogen synthesis protein glgS | -3.2 |
| *glk* | 1766398_s_at | b2388 | Glucokinase (EC 2.7.1.2) | -2.0 |
| *glnH* | 1766180_s_at | b0811 | Glutamine-binding periplasmic protein precursor | -2.5 |
| *glnP* | 1766676_s_at | b0810 | Glutamine transport system permease protein glnP | -2.6 |
| *glnQ* | 1759579_s_at | b0809 | Glutamine transport ATP-binding protein glnQ | -2.8 |
| *glpT* | 1761983_s_at | b2240 | Glycerol-3-phosphate transporter | -2.6 |
| *gltA* | 1762086_s_at | b0720 | Citrate synthase (EC 4.1.3.7) | -2.0 |
| *gltI* | 1761724_s_at | b0655 | Glutamate/aspartate periplasmic binding protein precursor | -3.1 |
| *gltJ* | 1768428_s_at | b0654 | Glutamate/aspartate transport system permease protein gltJ | -5.1 |
| *gltK* | 1765435_s_at | b0653 | Glutamate/aspartate transport system permease protein gltK | -4.0 |
| *gltL* | 1765895_s_at | b0652 | Glutamate/aspartate transport ATP-binding protein gltL | -2.8 |
| *gntP* | 1768471_s_at | b4321 | High-affinity gluconate transporter | -1.2 |
| *hcaR* | 1766136_s_at | b2537 | Hca operon transcriptional activator | -4.1 |
| *hdfR* | 1763167_s_at | b3762 | Possible regulatory protein pssR | -2.3 |
| *hexR* | 1767209_s_at | b1853 | Hex regulon repressor | -2.3 |
| *hokB* | 1763295_s_at | b4428 | homologue of the hok (host killing) gene | -1.8 |
| *katG* | 1767690_s_at | b3942 | Peroxidase/catalase HPI (EC 1.11.1.6) | -1.8 |
| *lamB* | 1760852_s_at | b4036 | Maltoporin precursor | -6.3 |
| *ldrD* | 1763059_s_at | b4453 | LdrD peptide of the LdrD-RdlD toxin-antitoxin system | -2.1 |
| *maeB* | 1767376_at | b2463 | NADP-dependent malic enzyme (EC 1.1.1.40) | -2.0 |
| *manX* | 1768684_s_at | b1817 | PTS system, mannose-specific IIAB component (EC 2.7.1.69) | -3.0 |
| *manY* | 1763069_s_at | b1818 | PTS system, mannose-specific IIC component | -2.9 |
| *manZ* | 1764762_s_at | b1819 | PTS system, mannose-specific IID component | -2.5 |
| *mdh* | 1766873_s_at | b3236 | Malate dehydrogenase (EC 1.1.1.37) | -1.8 |
| *melA* | 1765153_s_at | b4119 | Alpha-galactosidase (EC 3.2.1.22) | -2.2 |
| *melR* | 1763732_s_at | b4118 | Melibiose operon regulatory protein | -2.1 |
| *mglA* | 1762687_s_at | b2149 | Galactoside transport ATP-binding protein mglA | -3.0 |
| *mglB* | 1768444_s_at | b2150 | D-galactose-binding periplasmic protein precursor | -2.4 |
| *mglC* | 1761896_s_at | b2148 | Galactoside transport system permease protein mglC | -2.8 |
| *minD* | 1767749_s_at | b1175 | Septum site-determining protein minD | -1.8 |
| *minE* | 1766899_s_at | b1174 | Cell division topological specificity factor | -2.1 |
| *mokB* | 1762609_s_at | b1420 | Polypeptide: regulatory peptide whose translation enables hokB expression | -2.6 |
| *msrB* | 1767911_s_at | b1778 | Peptide methionine sulfoxide reductase msrB (EC 1.8.4.6) | -2.0 |
| *mtlD* | 1765898_s_at | b3600 | Mannitol-1-phosphate 5-dehydrogenase (EC 1.1.1.17) | -1.8 |
| *nanE* | 1766413_s_at | b3223 | Hypothetical protein yhcJ | -1.9 |
| *ompW* | 1763696_s_at | b1256 | Outer membrane protein W precursor | -2.3 |
| *omrA* | 1763092_at | b4444 | Regulatory-RNAs | -2.3 |
| *oppA* | 1764969_s_at | b1243 | Periplasmic oligopeptide-binding protein precursor | -1.8 |
| *oppB* | 1761726_s_at | b1244 | Oligopeptide transport system permease protein oppB | -4.0 |
| *osmC* | 1768689_s_at | b1482 | Osmotically inducible protein C | -3.1 |
| *osmY* | 1767463_s_at | b4376 | Osmotically inducible protein Y precursor | -2.8 |
| *pckA* | 1764813_s_at | b3403 | Phosphoenolpyruvate carboxykinase [ATP] (EC 4.1.1.49) | -1.9 |
| *pepE* | 1767176_s_at | b4021 | Peptidase E | -2.1 |
| *poxB* | 1762680_at | b0871 | Pyruvate dehydrogenase [cytochrome] (EC 1.2.2.2) | -2.7 |
| *psiF* | 1761076_s_at | b0384 | Phosphate starvation-inducible protein psiF precursor | -2.2 |
| *putA* | 1766150_s_at | b1014 | Bifunctional putA protein | -1.9 |
| *putP* | 1763553_s_at | b1015 | Sodium/proline symporter | -2.8 |
| *rbsA* | 1760971_s_at | b3749 | Ribose transport ATP-binding protein rbsA | -1.9 |
| *rbsC* | 1763403_s_at | b3750 | Ribose transport system permease protein rbsC | -1.9 |
| *rbsD* | 1763733_s_at | b3748 | High affinity ribose transport protein rbsD | -2.3 |
| *rbsK* | 1763275_s_at | b3752 | Ribokinase (EC 2.7.1.15) | -2.3 |
| *ribB* | 1766008_s_at | b3041 | 3,4-dihydroxy-2-butanone 4-phosphate synthase | -2.6 |
| *rof* | 1762109_s_at | b0189 | Rof protein | -2.1 |
| *rsd* | 1768012_s_at | b3995 | Regulator of sigma D | -2.8 |
| *ryeA* | 1759187_at | b4432 | Misc-RNAs | -2.0 |
| *sdhB* | 1769260_s_at | b0724 | Succinate dehydrogenase iron-sulfur protein (EC 1.3.99.1) | -2.2 |
| *sdhD* | 1767040_s_at | b0722 | Succinate dehydrogenase hydrophobic membrane anchor protein | -2.4 |
| *sodC* | 1767838_s_at | b1646 | Superoxide dismutase [Cu-Zn] precursor (EC 1.15.1.1) | -2.1 |
| *sokB* | 1759132_at | b4429 | Regulatory-RNAs | -2.7 |
| *sra* | 1767590_s_at | b1480 | 30S ribosomal protein S22 | -2.6 |
| *sraH* | 1766206_at | b4450 | Misc-RNAs | -3.7 |
| *srlA* | 1761750_s_at | b2702 | PTS system, glucitol/sorbitol-specific IIC2 component | -2.6 |
| *srlB* | 1764665_s_at | b2704 | PTS system, glucitol/sorbitol-specific IIA component (EC 2.7.1.69) | -2.3 |
| *ssnA* | 1761054_at | b2879 | SsnA protein | -3.5 |
| *sucD* | 1767719_s_at | b0729 | Succinyl-CoA synthetase alpha chain (EC 6.2.1.5) | -2.1 |
| *tktB* | 1762639_s_at | b2465 | Transketolase 2 (EC 2.2.1.1) | -2.5 |
| *tnaA* | 1761050_s_at | b3708 | Tryptophanase (EC 4.1.99.1) | -5.9 |
| *tnaB* | 1763204_s_at | b3709 | Low affinity tryptophan permease | -6.7 |
| *tnaC* | 1762126_s_at | b3707 | Tryptophanase leader peptide | -34.3 |
| *tsx* | 1766260_s_at | b0411 | Nucleoside-specific channel-forming protein tsx precursor | -2.6 |
| *ubiA* | 1765022_s_at | b4040 | 4-hydroxybenzoate octaprenyltransferase (EC 2.5.1.-) | -2.0 |
| *ugpA* | 1760060_s_at | b3452 | SN-glycerol-3-phosphate transport system permease protein ugpA | -1.9 |
| *uspB* | 1768250_s_at | b3494 | Universal stress protein B | -2.7 |
| *uspE* | 1762683_s_at | b1333 | Protein ydaA | -1.9 |
| *xdhB* | 1759957_s_at | b2867 | Xanthine dehydrogenase, FAD binding subunit (EC 1.1.1.204) | -2.0 |
| *yaaF* | 1760878_s_at | b0030 | Hypothetical protein yaaF | -3.9 |
| *yahK* | 1768802_s_at | b0325 | Hypothetical zinc-type alcohol dehydrogenase-like protein yahK | -2.1 |
| *yahN* | 1762866_s_at | b0328 | Hypothetical protein yahN | -3.6 |
| *yaiY* | 1768358_s_at | b0379 | Hypothetical protein yaiY | -2.2 |
| *yaiZ* | 1761079_s_at | b0380 | Hypothetical protein yaiZ | -1.7 |
| *ybaE* | 1764180_s_at | b0445 | Hypothetical protein ybaE | -3.5 |
| *ybaW* | 1766500_s_at | b0443 | Hypothetical protein ybaW | -3.6 |
| *ybaY* | 1767571_s_at | b0453 | Hypothetical protein ybaY precursor | -2.5 |
| *ybeK* | 1766971_s_at | b0651 | Hypothetical protein ybeK | -2.3 |
| *ybhQ* | 1761194_s_at | b0791 | Hypothetical protein ybhQ | -2.4 |
| *ybjP* | 1764790_at | b0865 | Putative lipoprotein ybjP precursor | -2.5 |
| *ycaC* | 1759749_s_at | b0897 | Protein ycaC | -3.7 |
| *yccJ* | 1769106_s_at | b1003 | Hypothetical protein yccJ | -3.6 |
| *ycgB* | 1765610_s_at | b1188 | Hypothetical protein ycgB | -3.0 |
| *ycgK* | 1763146_s_at | b1178 | Protein ycgK precursor | -2.4 |
| *yciI* | 1767224_s_at | b1251 | Protein yciI | -2.0 |
| *ydcL* | 1761191_s_at | b1431 | Hypothetical lipoprotein ydcL precursor | -1.7 |
| *ydcS* | 1764131_s_at | b1440 | Putative ABC transporter periplasmic binding protein ydcS precursor | -2.8 |
| *ydcU* | 1762984_s_at | b1442 | Hypothetical ABC transporter permease protein ydcU | -2.1 |
| *ydcW* | 1766377_s_at | b1444 | Putative betaine aldehyde dehydrogenase (EC 1.2.1.8) | -2.6 |
| *yddP* | 1763848_s_at | b1484 | Hypothetical ABC transporter ATP-binding protein yddP | -2.1 |
| *ydeN* | 1768132_s_at | b1498 | Putative sulfatase ydeN precursor (EC 3.1.6.-) | -2.5 |
| *yeaQ* | 1765823_s_at | b1795 | Hypothetical protein yeaQ | -2.1 |
| *yeaT* | 1759934_s_at | b1799 | Hypothetical transcriptional regulator yeaT | -2.1 |
| *yebV* | 1768481_s_at | b1836 | Hypothetical protein yebV | -4.3 |
| *yebW* | 1766893_s_at | b1837 | Hypothetical protein yebW | -1.7 |
| *yedX* | 1759346_s_at | b1970 | Transthyretin-like protein precursor | -1.8 |
| *yeeI* | 1764330_s_at | b1976 | Hypothetical protein yeeI | -3.9 |
| *yeiL* | 1766347_s_at | b2163 | Hypothetical protein yeiL | -1.9 |
| *yeiT* | 1763390_s_at | b2146 | Hypothetical oxidoreductase yeiT | -3.5 |
| *ygaM* | 1760727_s_at | b2672 | Hypothetical protein ygaM | -2.5 |
| *ygcO* | 1769277_s_at | b2767 | Ferredoxin-like protein ygcO | -1.9 |
| *ygdI* | 1766187_s_at | b2809 | Hypothetical lipoprotein ygdI precursor | -3.1 |
| *ygeV* | 1761113_s_at | b2869 | Hypothetical sigma-54-dependent transcriptional regulator ygeV | -2.1 |
| *ygfJ* | 1769032_s_at | b2877 | Hypothetical protein ygfJ | -2.9 |
| *yggE* | 1764974_s_at | b2922 | Hypothetical protein yggE | -1.8 |
| *yghZ* | 1766325_s_at | b3001 | Hypothetical protein yghZ | -2.8 |
| *ygjR* | 1760185_s_at | b3087 | Hypothetical oxidoreductase ygjR | -2.1 |
| *yhaH* | 1760548_s_at | b3103 | Hypothetical protein yhaH | -2.5 |
| *yhiP* | 1762148_s_at | b3496 | Hypothetical transporter yhiP | -2.1 |
| *yiaG* | 1764713_s_at | b3555 | Hypothetical protein yiaG | -1.7 |
| *yidF* | 1763622_s_at | b3674 | Hypothetical protein yidF | -2.2 |
| *yifE* | 1766633_s_at | b3764 | Protein yifE | -2.0 |
| *yihW* | 1760629_s_at | b3884 | Hypothetical transcriptional regulator yihW | -2.2 |
| *yjbJ* | 1765253_s_at | b4045 | Protein yjbJ | -2.9 |
| *yjcG* | 1763832_at | b4067 | Putative symporter yjcG | -5.1 |
| *yjdI* | 1769100_s_at | b4126 | Hypothetical protein yjdK | -2.7 |
| *yjdN* | 1768688_s_at | b4107 | PhnB protein | -2.4 |
| *yjgB* | 1765890_at | b4269 | Hypothetical zinc-type alcohol dehydrogenase-like protein yjgB | -2.5 |
| *yjjM* | 1761393_s_at | b4357 | Hypothetical protein yjjM | -4.6 |
| *yncB* | 1768211_s_at | b1449 | Putative NADP-dependent oxidoreductase yncB | -3.4 |
| *yniA* | 1762832_s_at | b1725 | Hypothetical protein yniA | -2.7 |
| *yodB* | 1767635_s_at | b1974 | Cytochrome b561 homolog 1 | -1.7 |
| *yohF* | 1760584_s_at | b2137 | Hypothetical oxidoreductase yohF | -1.9 |
| *yphA* | 1768189_s_at | b2543 | Hypothetical protein yphA | -3.1 |
| *yphF* | 1763073_s_at | b2548 | ABC transporter periplasmic binding protein yphF precursor | -4.9 |
| *yphG* | 1768058_s_at | b2549 | Hypothetical protein yphG | -4.6 |
| *yqeF* | 1768275_s_at | b2844 | Probable acetyl-CoA acetyltransferase (EC 2.3.1.9) | -2.4 |
| *yqjD* | 1764293_s_at | b3098 | Hypothetical protein yqjD | -1.9 |
| *yqjK* | 1762848_s_at | b3100 | Hypothetical protein yqjK | -2.1 |
